# IFN-**γ**R/STAT1 signaling in recipient hematopoietic antigen-presenting cells suppresses graft-versus-host disease

**DOI:** 10.1172/JCI125986

**Published:** 2023-02-01

**Authors:** Caisheng Lu, Huihui Ma, Liangsong Song, Hui Wang, Lily Wang, Shirong Li, Stephen M. Lagana, Antonia R. Sepulveda, Kasper Hoebe, Samuel S. Pan, Yong-Guang Yang, Suzanne Lentzsch, Markus Y. Mapara

**Affiliations:** 1 Columbia Center for Translational Immunology and; 2 Division of Hematology-Oncology, Columbia University, New York, New York, USA.; 3 Department of Pathology and Cell Biology, Columbia University, New York, New York, USA.; 4 Department of Pathology, George Washington University School of Medicine and Health Sciences, Washington DC, USA.; 5 Department of Pediatrics, University of Cincinnati, Cincinnati, Ohio, USA.; 6 Janssen Research and Development, Spring House, Pennsylvania, USA.

**Keywords:** Immunology, Transplantation, Antigen-presenting cells, Bone marrow transplantation, Tolerance

## Abstract

The absence of IFN-*γ* receptor (IFN-*γ*R) or STAT1 signaling in donor cells has been shown to result in reduced induction of acute graft-versus-host disease (GVHD). In this study, we unexpectedly observed increased activation and expansion of donor lymphocytes in both lymphohematopoietic organs and GVHD target tissues of IFN-*γ*R/STAT1–deficient recipient mice, leading to rapid mortality following the induction of GVHD. LPS-matured, BM-derived *Ifngr1^–/–^ Stat1^–/–^* DCs (BMDCs) were more potent allogeneic stimulators and expressed increased levels of MHC II and costimulatory molecules. Similar effects were observed in human antigen-presenting cells (APCs) with knockdown of *Stat1* by CRISPR/Cas9 and treatment with a JAK1/2 inhibitor. Furthermore, we demonstrated that the absence of IFN-*γ*R/STAT1 signaling in hematopoietic APCs impaired the presentation of exogenous antigens, while promoting the presentation of endogenous antigens. Thus, the indirect presentation of host antigens to donor lymphocytes was defective in IFN-*γ*R/STAT1–deficient, donor-derived APCs in fully donor chimeric mice. The differential effects of IFN-*γ*R/STAT1 signaling on endogenous and exogenous antigen presentation could provide further insight into the roles of the IFN-*γ*/STAT1 signaling pathway in the pathogenesis of GVHD, organ rejection, and autoimmune diseases.

## Graphical abstract

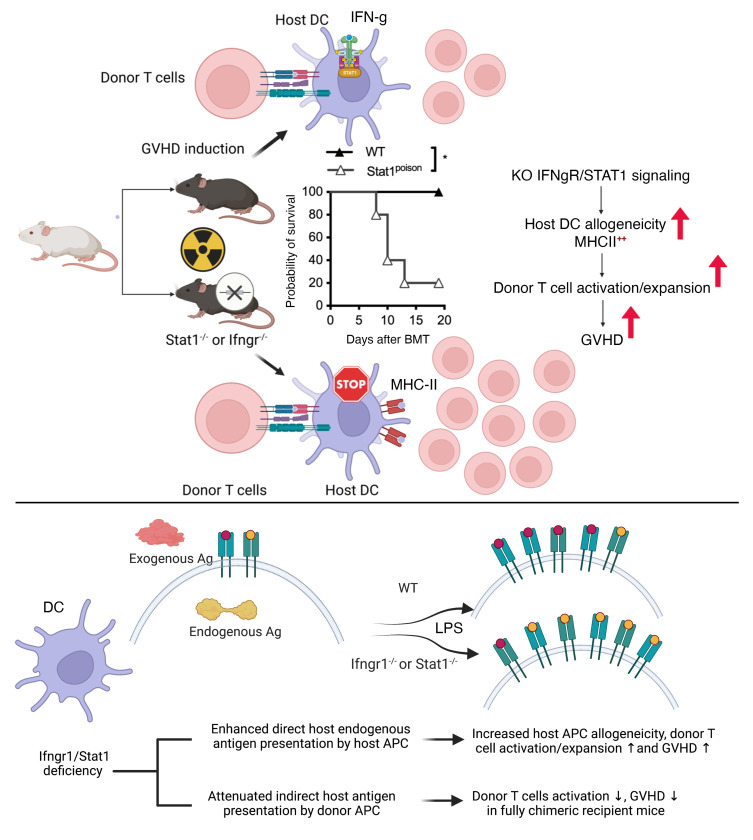

## Introduction

IFN-γ is a pleiotropic cytokine with a central role in host defense and immunopathology and is involved in both innate and adaptive immunity (1, 2). IFN-γ is the only type II IFN family member produced by innate NK, γδ-T cells, activated Th1 cells, cytotoxic CD8^+^ T cells, and professional antigen-presenting cells (APCs) (1–3). Acting synergistically with T cell receptor (TCR) stimulation as a prototypical Th1 cytokine, IFN-γ functions via the JAK1/STAT1 signaling pathway to drive the first wave of T-bet expression (4). T-bet serves as the master regulator of Th1 cell differentiation by promoting the expression of both IL-12 receptor β2 and the production of IFN-γ (4). This effect renders cells responsive to IL-12/STAT4 and IFN-γ/STAT1 to maintain T-bet expression and Th1-specific cytokine production, while inhibiting the differentiation and function of other Th cell subsets (4–6). In addition to its effects on Th cell differentiation, IFN-γ is known to promote macrophage activation (1, 2) and the expression of MHC class II and IL-12, which further amplify IFN-γ production and Th1-mediated physiologic and pathologic immune responses (1, 3, 7–12). IFN-γ signaling has been implicated in the mediation of tissue damage in the gastrointestinal (GI) tract (13, 14). Therefore, IFN-γR signaling is emerging as an attractive target (15–17) for graft-versus-host disease (GVHD) intervention, as corroborated by clinical results using the nonselective JAK1/2 inhibitor ruxolitinib (Rux) and preclinical results with barcitinib (18, 19).

Conversely, evidence supports a protective role for IFN-γ in limiting inflammation-driven tissue damage. In particular, *Ifng^–/–^*, *Ifngr1^–/–^*, and *Stat1^–/–^* mice are significantly more susceptible to experimental autoimmune encephalomyelitis (EAE) (20–22), collagen-induced arthritis (CIA) (12), and acute GVHD induction (8, 10, 23–25). This inhibitory function of IFN-γ has been proposed to involve the induction of apoptosis of activated lymphocytes (26, 27), the conversion of CD4^+^CD25^–^ T cells into Tregs (28), and the induction of indoleamine 2,3-dioxygenase (IDO) and NO expression leading to tolerogenic DCs (1–3, 26). Furthermore, IFN-γ–induced programmed death ligand 1 (PD-L1) expression negatively regulates T cell activation (29).

Here, we describe a modulatory role for IFN-γ/STAT1 signaling in hematopoietic APCs. We observed markedly enhanced GVHD induction and increased activation of alloreactive donor T cells by host IFN-γR/STAT1–deficient hematopoietic APCs. The enhanced stimulatory capacity of IFN-γR/STAT1–deficient APCs was associated with the upregulation of MHC II expression and increased endogenous antigen presentation. Radiation bone marrow (BM)chimeric recipients with fully engrafted donor-derived IFN-γR–deficient APCs had less indirect allostimulatory capacity, resulting in attenuated activation of host-alloantigen–specific donor lymphocytes from the delayed donor lymphocyte infusion (DLI) and development of GVHD. Our data suggest that IFN-γR/STAT1 signaling in APCs functions as an immune rheostat to restrain autoinflammation by suppressing endogenous antigen presentation under inflammatory conditions while enhancing responses against exogenous antigens.

## Results

### Absence of the IFN-γR/STAT1 signaling in recipient mice leads to increased acute GVHD.

**Figure 1:**
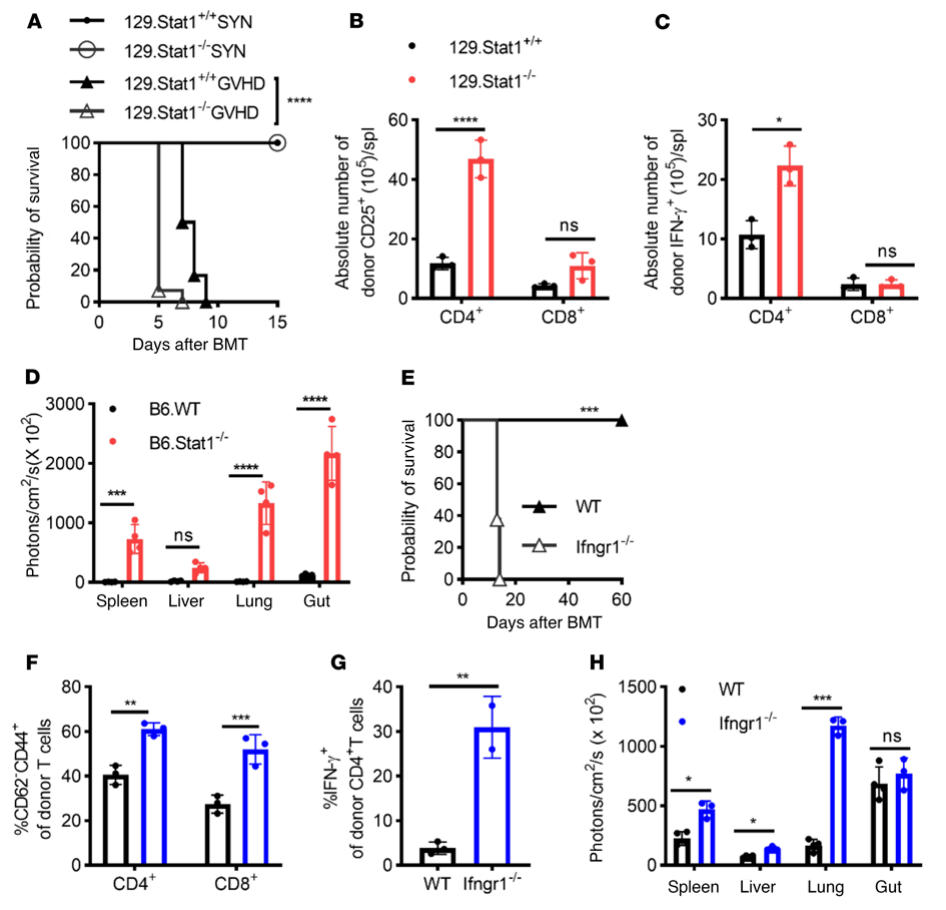
Absence of host IFN-γR/STAT1 signaling enhances GVHD induction. GVHD was induced in the fully MHC-mismatched [BALB/c (H2^d^) to 129Sv (H2^b^)] strain combination. SYN, syngeneic transplant. (**A**) Lethally irradiated (1,044 cGy) 129.*Stat1^–/–^* or 129.*Stat1^+/+^* mice received 5 × 10^6^ BMCs and 1 × 10^7^ SPCs from BALB/c mice. Data are from 2 similar experiments with 12–14 animals/group. (**B** and **C**) CD25 expression (**B**) and intracellular IFN-γ expression (**C**) on donor (H2D^d^) CD4^+^ or CD8^+^ T cells in the host spleen were tested on day 4 after BMT. Representative results from 1 of 2 independent experiments with 3–4 animals/group are shown. (**D**) In vivo expansion of alloreactive donor BALB/c-Luc T cells and target organ infiltration in B6.WT or *Stat1^–/–^* recipients were assessed by BLI on post-BMT day 6. Representative results from 1 of 3 independent experiments with 3–4 animals per group are shown. (**E**–**H**) Fully MHC-mismatched GVHD induction following lethal irradiation in B6 (WT, *n* = 5) versus B6.*Ifngr1^–/–^* recipients (*Ifngr1^–/–^*, *n* = 8) using 1 × 10^7^ SPCs and 5 × 10^6^ BMCs from BALB/c-Luc mice. Survival data were analyzed by log-rank test. Activation of donor (H2^d+^) CD4^+^ T cells, CD8^+^ T cells, and lymphocytes and (**F**) intracellular IFN-γ staining of donor-derived CD4^+^ T cells (**G**) was tested on day 4 after BMT. (**H**) Recipient animals were monitored for infiltration and expansion of BALB/c-Luc lymphocytes on day 7 after BMT using BLI. Representative results from 1 of 2 independent experiments with 3–4 animals/group are shown. Bar graphs represent the mean ± SEM. *P < 0.05, **P < 0.01, ***P < 0.001, and ****P < 0.0001, by log-rank test (**A**), 2-way ANOVA with Šidák’s correction (**B**–**F** and **H**), and Student’s *t* test (**G**).

In the current study, we sought to investigate the effects of host STAT1 deficiency on the induction of acute GVHD. To this end, we induced GVHD in the fully MHC-mismatched BALB/c (H2^d^) to 129 (H2^b^) strain combination by injecting 5 × 10^6^ BALB/c T cell–depleted (TCD) BM cells (BMCs) plus 1 × 10^7^ splenocytes (SPCs) into lethally irradiated 129.*Stat1^+/+^* or 129.*Stat1^–/–^* mice. The 129.*Stat1^–/–^* recipients experienced significantly accelerated mortality (Figure 1A, median survival time [MST] of 5 days vs. 7.5 days, log-rank test *P* < 0.0001) and exacerbated morbidity, as measured by GVHD clinical scores (Supplemental Figure 1A; supplemental material available online with this article; https://doi.org/10.1172/JCI125986DS1). However, histopathological analysis of GVHD target organs obtained on day 4 showed no significant differences yet at this early time point between the WT and STAT1-deficient recipients (Supplemental Figure 1B). STAT1-deficient hosts receiving syngeneic BMCs and splenic cells did not exhibit increased morbidity or mortality, ruling out the possibility that enhanced sensitivity of STAT-deficient recipients to conditioning-induced toxicity accounted for the increased mortality. To investigate the inflammatory response following allogeneic BM transplantation (BMT), we tested the early serum cytokine profiles of the recipient mice after lethal irradiation following BMT. Our data did not reveal increased serum levels of IL-1α, IL-6, TNF-α, RANTES, or monocyte chemoattractant protein 1 (MCP-1) on day 1 and day 3 after BMT in the *Stat1^–/–^* group. However, we observed significant increases in IL-12 and IL-4 on day 3 and/or day 4 after BMT in the *Stat1^–/–^* recipient mice compared with their WT counterparts (Supplemental Figure 2), indicating dysregulation and skewing of the immune response. When recipient splenic cells were analyzed by flow cytometry on day 5 after BMT, we found increased expansion of donor T cells and elevated absolute numbers of activated donor CD4^+^ T cells (Figure 1B). The increase was associated with enhanced donor CD4^+^ Th1 but comparable CD8^+^ Tc1 cell differentiation in *Stat1^–/–^* recipients (Figure 1C).

Using bioluminescence imaging (BLI), we tested in vivo expansion of donor cells and organ infiltration after the transfer of luciferase-expressing BALB/c SPCs (BALB/c-Luc) into lethally irradiated B6.*Stat1*-deficient and B6 WT recipient mice. B6 mice are more resistant to GVHD induction than the 129sv mice under the same transplantation conditioning, which allowed us to observe donor lymphocyte expansion after post-BMT day 5. Markedly enhanced expansion and organ infiltration of donor BALB/c-Luc cells were detectable in the spleen, liver, lung, and gut of B6.*Stat1^–/–^* mice compared with B6 WT recipients (Figure 1D and Supplemental Figure 3A) on day 6 after BMT. We repeated our studies in a different STAT1-deficient strain (B6.*Stat1^Poison^* mice) to further validate our results. This mouse strain was generated by *N*-ethyl-*N*-nitrosourea–induced (ENU-induced) mutagenesis, which affects the splice site upstream of exon 20 of the *Stat1* gene, resulting in a truncated STAT1 protein (30). The phenotypes of STAT1^Poison^ mice and 129.STAT1-deficient mice were consistent with those of mice in our prior study (17) (Supplemental Figure 4A and data not shown). In line with experiments performed with *129.* *Stat1^–/–^* mice, GVHD-induced mortality significantly increased in *Stat1^Poison^* recipients (Supplemental Figure 4B). CD4^+^ and CD8^+^ T cell activation was also enhanced (Supplemental Figure 4C). Similar to the results published by Burman et al. in *Ifngr1^–/–^* mice (14), we noted severe lung pathology (data not shown) but reduced damage to the GI tract in *Stat1^Poison^* recipient mice when histopathological analysis was performed on day 8 after BMT (Supplemental Figure 4, D and E). These results confirm the differential role of IFN-γR signaling in GVHD target organs (13).

We next delineated the contribution of IFN-γ signaling in recipient mice using the BALB/c B6 model and the ability of IFN-γ signaling deficiency to recapitulate our STAT1 findings. Following the observation that GVHD-related mortality was significantly higher in Stat1*^–/–^* recipient mice than in the WT recipients, GVHD induction was accelerated considerably (MST of 13 days in the *Ifngr1^–/–^* versus undefined in the WT group, Figure 1E) in the B6.*Ifngr1^–/–^* recipients following lethal irradiation (10.75 Gy). This observation was consistent with the studies reported by Burman and colleagues (14). We observed enhanced activation and expansion of donor CD4^+^ and CD8^+^ T cells (Figure 1F) and significantly increased Th1 differentiation of donor CD4^+^ lymphocytes (Figure 1G) in the spleens of the *Ifngr1^–/–^* recipient mice when tested on day 4 or 5 after BMT. Using BLI analysis of recipients injected with BALB/c-Luc splenic cells, we also observed significantly enhanced donor lymphocyte infiltration in the gut and spleen, tested on day 4 after BMT (Supplemental Figure 5B) in the B6.*Ifngr1^–/–^* recipients compared with the B6 WT recipients. When studied on day 7, we observed a further increase in tissue infiltration (spleen, lung, and liver) of donor lymphocytes in B6.*Ifngr^–/–^* recipients. In contrast to the *Stat1^–/–^* recipients, donor lymphocyte infiltration in the gut of B6.*Ifngr1^–/–^* recipients was comparable to that of their WT counterparts when assessed on day 7 (Figure 1H and Supplemental Figure 3B). These results suggest that the lack of IFN-γ signaling accelerated the initial gut infiltration but did not result in further accumulation at later time points. Furthermore, our results indicate different roles in host STAT1 and IFN-γR signaling regarding the recruitment of donor cells to the gut, which warrants further study.

### Contribution of hematopoietic versus nonhematopoietic IFN-γR/STAT1 deficiency to the development of GVHD.

**Figure 2:**
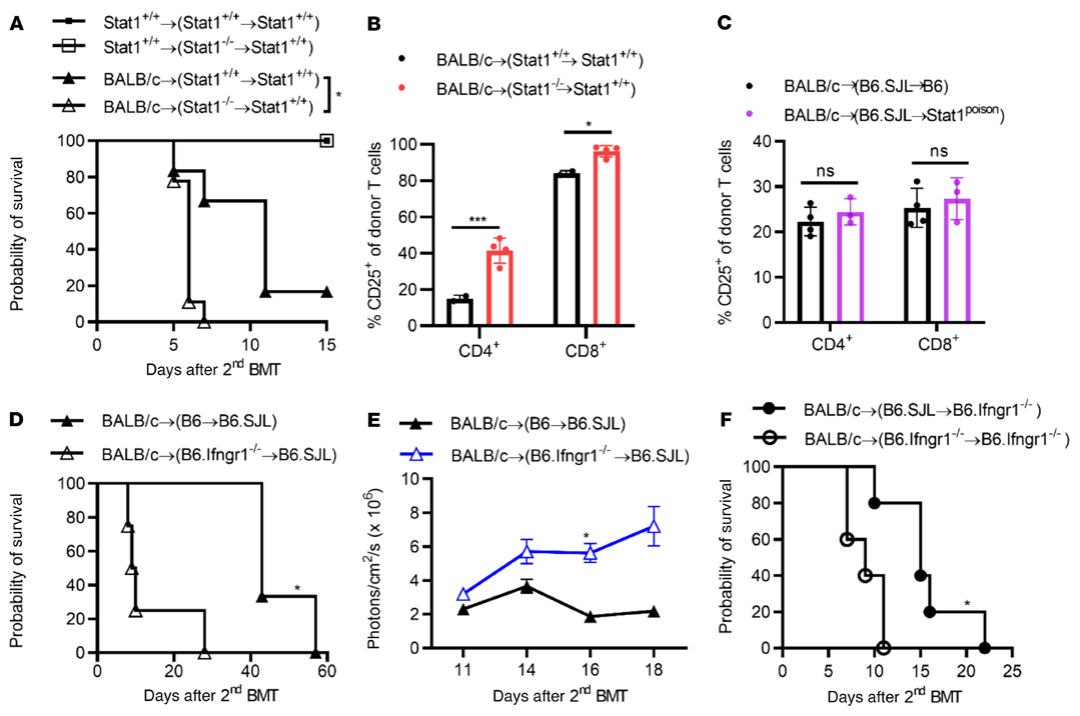
Contribution of hematopoietic versus nonhematopoietic IFN-γR/STAT1 deficiency to the development of GVHD. (**A** and **B**) GVHD was induced in radiation chimeras with STAT1 deficiency in the hematopoietic compartment (129.*Stat1^–/–^*→129.*Stat1^+/+^*). The data are representative of 2 similar experiments with 9–12 mice/group. (**A**) MST was 6 days versus 11 days (*P* = 0.02, log-rank test). (**B**) CD25 expression on donor (H-2^d^) CD4^+^ and CD8^+^ T cells was assessed on day 6 after BMT. (**C**) Donor lymphocyte activation was assessed in chimeric mice with STAT1 deficiency restricted to nonhematopoietic cells using B6.*Stat1^Poison^* mice. Lethally irradiated (1,075 cGy) B6.*Stat1^Poison^* mice or B6 WT mice received 5 × 10^6^ BMCs from B6.SJL syngeneic WT mice. Four months after the first transplantation, the chimeras were irradiated (1,075 cGy) and injected with 5 × 10^6^ BMCs and 1 × 10^7^ splenocytes from BALB/c mice. CD25 expression on donor (H-2^d^) CD4^+^ and CD8^+^ T cells was measured on day 7 after BMT (*n* = 5 mice/group). (**D**–**F)** GVHD induction in irradiated chimeras with IFN-γR deficiency in the hematopoietic (B6.*Ifngr1^–/–^*→B6.SJL) (**D** and **E**) and nonhematopoietic compartments (B6.SJL→B6.*Ifngr1^–/–^*) (**F**). (**D**) Probability of survival (MST of 9.5 days vs. 43 days, log-rank test *P < 0.05, *n* = 3–6 mice/group). (**E**) In vivo expansion of BALB/c-Luc lymphocytes in recipient animals was monitored using BLI on the indicated days after the second transplantation. (**F**) Survival curve following GVHD induction in *Ifngr1^–/–^*→*Ifngr1^–/–^* chimeras with BALB/c splenic cells (MST of 15 days vs. 9 days, log-rank test *P* < 0.05, *n* = 5–6 mice/group). Bar graphs represent the mean ± SEM. *P < 0.05 and ***P < 0.001, by 2-way ANOVA with Šidák’s correction.

To determine the contribution of host hematopoietic versus nonhematopoietic tissue to the promotion of GVHD, we created radiation BM chimeric mice, in which STAT1 deficiency was confined to the hematopoietic compartment, and then induced GVHD via a second transplant. Thus, 129*.Stat1^+/+^* mice were lethally (10.44 Gy) irradiated and reconstituted with either 129*.Stat1^+/+^* or *Stat1^–/–^* BMCs. The successful creation of radiation chimeras and the absence of STAT1 expression were confirmed by STAT1 staining in peripheral blood samples using flow cytometry (data not shown). On day 120 after BMT, the mice were reconditioned (10.44 Gy) and transplanted with 5 × 10^6^ BMCs plus 5 × 10^6^ splenic cells from BALB/c mice. As shown in Figure 2A, we observed accelerated GVHD-induced mortality in BALB/c→(129*.Stat1^–/–^*→129*.Stat1^+/+^*) chimeric mice compared with BALB/c→ (129*.Stat1^+/+^*→*Stat1^+/+^*) chimeras (MST of 6 days vs. 11 days, log-rank *P* = 0.02), suggesting that the absence of STAT1 in host hematopoietic tissue was sufficient to accelerate GVHD induction. Neither the *Stat1^–/–^* mice nor the WT hosts receiving syngeneic BMCs and splenic cells died, confirming that the alloreactive responses were the driving force of acute GVHD induction in this model. In accordance with the increased mortality, donor T cell activation was significantly increased in BALB/c→(129.*Stat1^–/–^*→129*.Stat1^+/+^)* recipients tested on day 6 after BMT (Figure 2B). To further confirm that the increased activation of donor lymphocytes was indeed triggered by STAT1-deficient host hematopoietic cells (and not by nonhematopoietic cells), we established similar chimeras using Β6.*Stat1^Poison^* mice. Β6.*Stat1^Poison^* or WT (*Stat1^+/+^*) B6 mice (both CD45.2) were lethally (10.75 Gy) irradiated and reconstituted with B6.SJL (CD45.1) BMCs before undergoing a second transplantation for GVHD induction, as described above. In contrast to the chimeric mice with exclusive hematopoietic STAT1 deficiency, we found that donor T cell activation was not increased in the Β6.*Stat1^+/+^*→*Stat1^Poison^* chimeric recipient mice upon induction of GVHD following retransplantation compared with that observed in the Β6.*Stat1^+/+^*→*Stat1^+/+^* chimeric mice (Figure 2C). These results suggest that the absence of STAT1 in nonhematopoietic host tissue was not critical to affecting the initial donor T cell activation, whereas STAT1 deficiency in the recipient hematopoietic cells was sufficient for enhanced donor T cell activation and subsequent morbidity and mortality.

Next, we determined the contribution of host hematopoietic versus nonhematopoietic IFN-γR deficiency in promoting GVHD. We created radiation BM chimeric mice (Β6.*Ifngr1^–/–^*→B6.SJL), in which IFN-γR deficiency was confined to the hematopoietic compartment, as described above. After reconditioning and the induction of GVHD [BALB/c→(Β6.*Ifngr1^–/–^*→B6.SJL]], we observed increased GVHD-induced mortality (Figure 2D) and enhanced expansion of infused donor BALB/c-Luc lymphocytes in recipient chimeric mice lacking the IFN-γR on host hematopoietic cells, as tested by BLI (Figure 2E). Recipient nonhematopoietic IFN-γR signaling had been reported to play an important role in reducing GVHD-induced mortality (31). Our data also support this observation, with a MST of 43 days (solid triangle in Figure 2D) in recipient mice with IFN-γR deficiency restricted to the nonhematopoietic cells versus a MST of 15 days (solid circle in Figure 2F) in chimeric mice with intact IFN-γR in both hematopoietic and nonhematopoietic compartments (Figure 2, D and F). Of note, compared with recipients deficient in IFN-γR expression only in nonhematopoietic tissue, GVHD-induced mortality was further enhanced when both hematopoietic and nonhematopoietic tissues (BALB/c→(B6.*Ifngr1^–/–^*→Β6.*Ifngr1^–/–^*) lacked IFN-γR (Figure 2F). These data suggest that IFN-γR/STAT1 signaling in the host hematopoietic compartments plays a vital role in reducing the activation of donor lymphocytes and the induction of acute GVHD in the MHC-mismatched BMT mouse model.

### Phenotypic and functional characteristics of IFN-γR/STAT1–deficient APCs.

**Figure 3:**
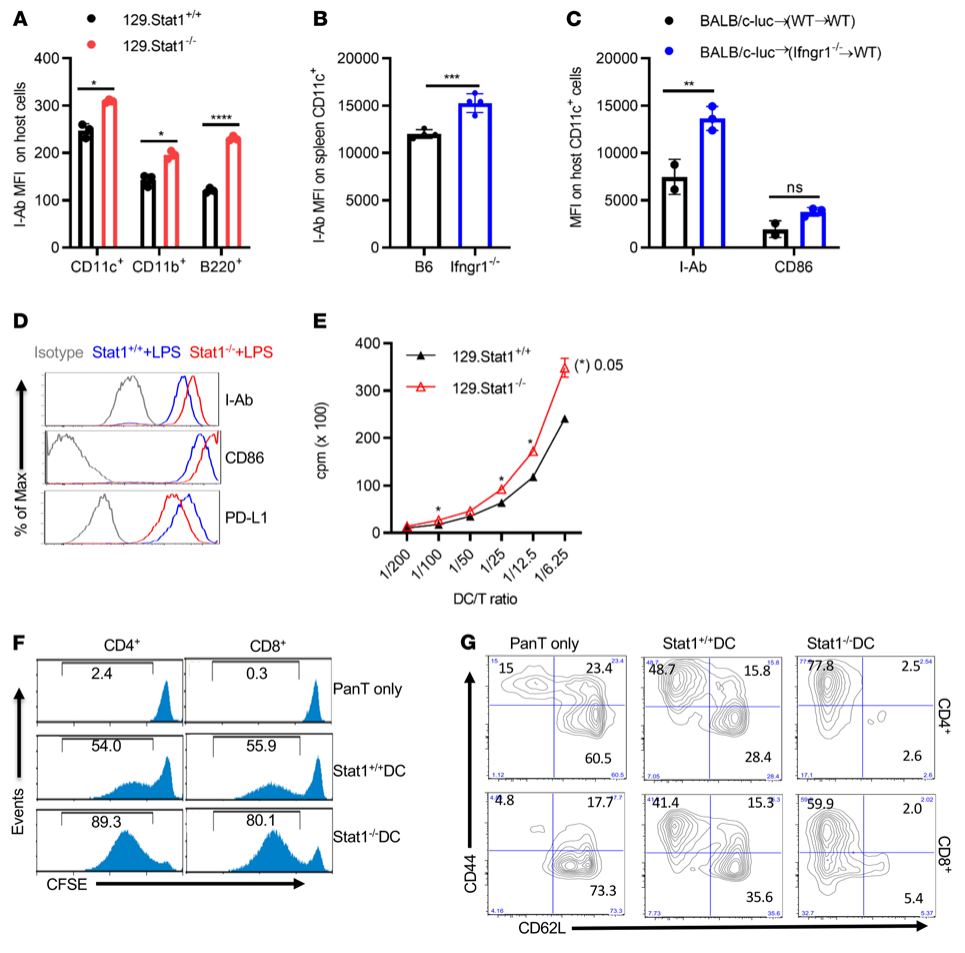
Absence of IFN-γR/STAT1 promotes an allostimulatory capacity in DCs. (**A**) On day 1 after BMT, different APC populations from recipient animals were analyzed following induction of GVHD using a fully MHC-mismatched [BALB/c (H2^d^) to 129Sv (H2^b^)] strain combination. MHC II expression on recipient splenic H2^b+^ CD11c^+^, CD11b^+^, and B220^+^ cells from recipient 129.*Stat1*^+/+^ and 129*. Stat1^–/–^* animals. Results from 1 representative experiment of 3 are shown, with 3 animals/group. (**B**) GVHD was induced in the fully MHC-mismatched BALB/c (H2^d^) to B6 (H2^b^) strain combination in B6 WT or B6.*Ifngr1^–/–^* mice. I-A^b^ expression on recipient splenic CD11c^+^ cells was studied on day 1 after BMT. Results from 1 representative experiment of 2 are shown with 3–4 animals/group. (**C**) I-Ab and CD86 expression on CD11c^+^ cells from *Ifngr1^–/–^*→B6.SJL chimeric recipients were compared with expression on cells from the B6→B6.SJL counterparts 2 days after the second transplantation of BALB/c TCD BMCs and BALB/c-Luc splenocytes. Results from 1 representative experiment of 2 are shown, with 2–3 animals per group. (**D**) Expression of I-Ab, CD86, and PD-L1 in CD11c^+^ BMDCs from *Stat1^+/+^* or *Stat1^–/–^* mice. Cells were cultured in RPMI 1640 medium containing 10% FCS and GM-CSF (20 ng/mL) for 6 days and matured in 100 ng/mL LPS for an additional 48 hours. Results from 1 representative experiment of more than 3 independent experiments are shown. (**E**–**G)** Proliferation and activation of CFSE-labeled alloreactive pan–T cells isolated from BALB/c splenocytes stimulated with LPS-matured *Stat1^+/+^* or *Stat1^–/–^* BMDCs. Freshly isolated pan–T cells from BALB/c mice cells were stimulated for 5 days with LPS-matured *Stat1^+/+^* or *Stat1^–/–^* BMDCs at a DC/responder ratio of 1:5. The proliferation of responder cells was assessed by ^3^H-incorporation, presented as the cpm ratio compared with unstimulated responder cells (**E**), or by CFSE dilution, presented as the percentage of CFSE^lo^ population (**F**). T cell activation was measured by CD44 and CD62L expression in responder CD4^+^ or CD8^+^ T cells (**G**). Results from 1 representative experiment of 3 independent experiments are shown. Bar graphs represent the mean ± SEM. *P < 0.05, **P < 0.01, ***P < 0.001, and ****P < 0.0001, by 2-way ANOVA with Šidák’s correction.

Next, we investigated the impact of IFN-γR/STAT1 deficiency on the hematopoietic APC phenotype and function. To our surprise, MHC II surface expression levels were significantly elevated on recipient IFN-γR/STAT1–deficient APCs compared with levels in their WT counterparts, as tested on days 1–3 after BMT in the MHC-mismatched mouse GVHD model (Figure 3, A and B). Our radiation chimera model further corroborated these results. Thus, surface expression of MHC II and CD86 was consistently higher on host splenic CD11c^+^ cells in recipients lacking IFN-γR only in the hematopoietic compartment (BALB/c→[B6.*Ifngr1^–/–^*→B6.SJL]) compared with WT chimeric (BALB/c→[B6 →B6.SJL]) controls (Figure 3C).

To further investigate the increased expression of MHC II and costimulatory molecules on APCs with IFN-γR/STAT1 deficiency, we interrogated BM-derived DCs (BMDCs) isolated from either *Stat1^+/+^* or S*tat1^–/–^* mice for expression of MHC II and costimulatory molecules and their stimulatory capacity. LPS is one of the most commonly used reagents for BMDC maturation and a key inflammatory factor driving GVHD induction after lethal conditioning (32). Consistent with the in vivo observations, STAT1-deficient BMDCs matured in the presence of LPS expressed increased MHC II and CD86 (Figure 3D) on the cell surface compared with their WT counterparts. In contrast, expression of the coinhibitory molecule PD-L1 was reduced upon LPS treatment (Figure 3D) in STAT1-deficient BMDCs. Analysis of flow data from 6 separate in vitro experiments of LPS-matured DCs showed a substantial increase in I-Ab expression on *Stat1^–/–^* DCs compared with *Stat1^+/+^* DCs. Upregulation of CD86 and downregulation of PD-L1 expression on *Stat1^–/–^* DCs matured with LPS were consistently observed in 4 and 3 experiments, respectively, but did not reach statistical significance (data not shown).

Given the increased MHC II and CD86 expression levels, matured STAT1-deficient DCs would be expected to have an increased allostimulatory capacity. To test this hypothesis, irradiated LPS-matured *Stat1^+/+^* or *Stat1^–/–^* BMDCs were cocultured with fully MHC-mismatched (BALB/c) T cells at 1:5 T/DC ratios for 5 days. We were able to observe an enhanced proliferation of responder alloreactive T cells (Figures 3, E and F) and a markedly increased proportion of activated (CD44^+^CD62L^–^) CD4^+^ and CD8^+^ T cells (Figure 3G).

To determine the translational relevance of our above-described observations in mice, we tested the effect of deficient IFN-γR/STAT1 signaling on the function and phenotype of human APCs. We used *Stat1* CRISPR-directed gene editing in freshly isolated PBMCs. ATTO-550^+^ cells were collected with an Influx sorter, irradiated, and then used to stimulate alloreactive human PBMCs in a mixed lymphocyte reaction (MLR) assay (Supplemental Figure 6A). As expected, we detected no proliferation (determined by CellTracer Violet low) or activation (determined by HLA-DR^+^CD38^+^ expression on CD4^+^ or CD8^+^ T cells) of responder cells in the absence of stimulator cells. Control sgRNA-transduced stimulator cells induced 40% proliferation and 10% activation of responder cells. In contrast, enhanced proliferation (60%) and activation (15%) of responder cells were observed when *Stat1* sgRNA–treated stimulator cells were used (Supplemental Figure 6B). Next, we tested the effect of pharmacological inhibition of JAK1 and JAK2, which are upstream of STAT1 signaling. Rux is a JAK1/JAK2 inhibitor that was recently FDA approved for steroid-refractory GVHD. To delineate whether JAK inhibition could mimic the data observed in gene-deficient murine APCs, we performed additional studies using human CD3- and CD56-depleted PBMCs treated with 5 μM Rux (Supplemental Figure 7A) as stimulator cells. In contrast to a previous report that showing Rux inhibited DC activation and function (33), our data demonstrated that Rux pretreatment of CD3- and CD56-depleted PBMCs led to pronounced inhibition of PD-L1 expression, while promoting HLA-DR and CD86 expression on CD11c^+^ cells (Supplemental Figure 7B) associated with increased allostimulatory capacities in a MLR assay (Supplemental Figure 7C). The discrepancy with the published results may be partially due to our experimental system, which included T lymphocyte and NK cell depletion. In addition, Heine et al. used plastic adherence to generate monocyte-derived DCs, which may not necessarily deplete lymphocytes. Rux is well known for its inhibition of T and NK cell functions. Its influence on the purified APC population, including DCs, warrants further investigation.

### Effect of IFN-γR/STAT1 signaling on endogenous and exogenous antigen presentation.

Activation of donor CD4^+^ T cells requires a cognate interaction of their TCR with allopeptides presented within the context of MHC II molecules by the host or donor APCs (31, 34). Alloreactive donor CD4^+^ T cells can recognize host alloantigens in the context of MHC II via a direct or indirect pathway (34). In the direct pathway, donor T cells recognize host allopeptides in the context of intact host MHC molecules on the surface of recipient APCs. In the indirect pathway, donor T cells recognize host-derived allopeptides taken up, processed, and presented by MHC II on donor APCs. Therefore, we assessed the effect of IFN-γR/STAT1 signaling on the presentation of exogenous versus endogenous peptides in the context of MHC II.

**Figure 4:**
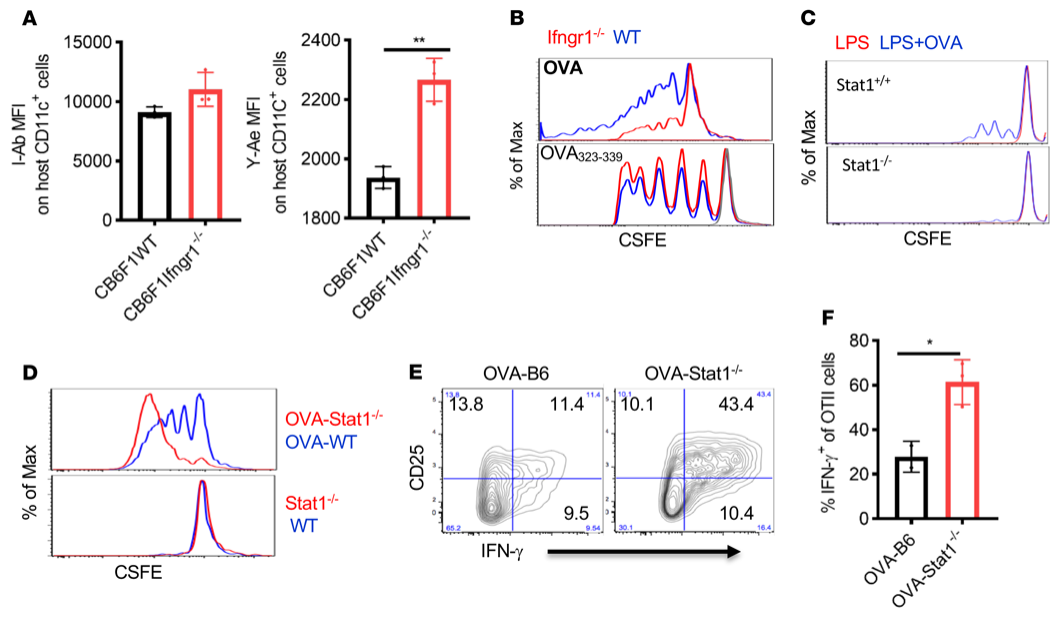
Absence of IFN-γR/STAT1 signaling leads to enhanced endogenous and compromised exogenous antigen presentation. (**A**) GVHD was induced in the parent-into-F1 (P→F1) mouse model without irradiation. CB6F1 (H2^bd^) WT or *Ifngr1^–/–^* mice received 5 × 10^6^ BMCs and 2.5 × 10^7^ to 3 × 10^7^ splenocytes from B6.SJL mice. There was an increase in I-Ab expression and recipient-derived endogenous Ea52-68 peptide presentation (tested by Y-Ae staining) on CD11c^+^ cells on day 2 after BMT. Data are representative of 2 similar experiments with 5 animals/group. (**B** and **C)** OT-II T cell proliferation was determined by CFSE dilution in response to 3 days of stimulation with 0.5% PFA-fixed B6.*Ifngr1^–/–^* or *Stat1^–/–^* BMDCs. BMDCs were incubated for 1 hour with OVA (100 ng/mL) and then overnight in the presence of LPS. For OVA_323–339_ peptide loading, BMDCs were matured with LPS, fixed with 0.5% PFA, and then pulsed with 100 ng/mL OVA_323–339_ peptides for 0.5 hours. (**D**) OT-II proliferation in response to 3 days of stimulation with WT act-mOVA (OVA-B6) or *Stat1^–/–^* act-mOVA (OVA-*Stat1^–/–^)* BMDCs after overnight LPS maturation. WT and *Stat1^–/–^* BMDCs without constitutive OVA expression were used as controls. (**E** and **F**) OVA-B6 or OVA-*Stat1^–/–^* mice were lethally irradiated (10.75 Gy) and received 3 × 10^6^ splenocytes from OTII mice. CD25 and IFN-γ expression was detected in OT-II cells 5 days after injection. *n* = 2–3 mice/group. **F** shows summary data for **E**. Bar graphs in **A**, **E**, and **F** represent the mean ± SEM. *P < 0.05 and **P < 0.01, by 2-tailed Student’s *t* test. Max, maximum.

Given the increased MHC II expression observed on host hematopoietic APCs lacking IFN-γR or STAT1 during the induction of GVHD, we assessed the association between enhanced MHC II expression and the increased presentation of host-derived endogenous peptides. The semiallogeneic B6.SJL→CB6F_1_ GVHR model without conditioning allowed us to identify self-peptide presentation by host APCs and subsequent antigen-specific donor T cell responsiveness. CB6.F1 mice express BALB/c-derived MHC class II Eα self-peptide (peptide 52–68), which is presented by I-A^b^ molecules, and this Eα–I-A^b^ complex is recognized by both the Y-Ae antibody and the TEa TCR (31, 35). For this experiment, 2.5 × 10^7^ to 3 × 10^7^ SPCs from B6.SJL mice and 5 × 10^6^ TCD BMCs from B6 mice were injected into either WT or *Ifngr1^–/–^* CB6F1 mice without irradiation. According to the above data, the absence of IFN-γR expression on CB6F1 recipient APCs promoted the expression of MHC class II (I-A^b^) on host CD11c^+^ cells compared with CB6F1 WT recipients following the administration of semiallogeneic B6 splenic cells tested on post-transplantation day 1. Y-Ae binding on host CD11c^+^ cells from CB6F1 *Ifngr1^–/–^* recipients was significantly increased (Figure 4A).

Next, we confirmed that the enhanced Y-Ae expression on host APCs in *Ifngr1^–/–^* CB6F1 mice elicited an enhanced Eα52-68 peptide–specific donor T cell response. To this end, TEa-TCR–Tg CD4^+^ T cells were coadministered to CB6F1 *Ifngr1^–/–^* or WT CB6F1 mice. As hypothesized, we observed markedly increased donor Tea-TCR^+^CD4^+^ T cell activation (Supplemental Figure 8A), Th1 differentiation (Supplemental Figure 8B), reduced Treg differentiation (Supplemental Figure 8C), and increased T cell proliferation as determined by BrdU incorporation (Supplemental Figure 8D) in CB6F1 *Ifngr1^–/–^* recipients. These findings suggested that the absence of IFN-γR/STAT1 signaling in recipient APCs enhanced host MHC II–dependent presentation of Eα52-68 and promoted recognition by donor TEa-TCR^+^CD4^+^ T cells.

The absence of IFN-γR or STAT1 in macrophages or DCs has been reported to result in defective antigen presentation for intracellular pathogens (36–39). To more clearly assess the effect of IFN-γR/STAT1 signaling on the presentation of exogenous antigens, we used OVA as an additional model antigen system. We studied the proliferation and activation of responder CD4^+^ OT-II cells as a readout for effective antigen presentation in APCs in the presence or absence of IFN-γR/STAT1 signaling. As expected, OT-II cells did not proliferate when cocultured with BMDCs matured by LPS without exogenous OVA protein (Figure 4C, red line). At the same time, the ability of IFN-γR– or STAT1-deficient BMDCs incubated with exogenous full-length OVA protein to promote OT-II proliferation was severely compromised (Figure 4B, upper panel, and Figure 4C, blue line). In contrast, OT-II proliferation was not diminished when stimulated with IFN-γR–deficient BMDCs that had been loaded with OVA_323–339_ peptide (Figure 4B, lower panel), which does not require intracellular antigen processing and presentation but depends on surface MHC II expression, indicating that direct loading of the peptide onto the MHC II peptide–binding groove was not impaired.

We next used transgenic act-mOVA mice, which have constitutive membrane-associated OVA expression in all tissues under the control of the actin promoter, as a model of the endogenous self-peptide presentation via MHC II. When these LPS-matured, mOVA-expressing, and STAT1-deficient BMDCs were used as stimulators, we observed markedly increased OT-II cell proliferation in response to *Stat1^–/–^* act-mOVA–expressing BMDCs compared with WT act-mOVA–expressing BMDCs (Figure 4D, upper panel), suggesting that the absence of STAT1 signaling promoted the MHC II–dependent presentation of OVA self-peptides. In contrast, we observed no OT-II cell proliferation in response to either WT or *Stat1^–/–^* BMDCs, confirming that the OT-II proliferation was OVA specific (Figure 4D, lower panel). The activation and Th1 differentiation of OT-II cells was markedly enhanced on day 3 after injection into lethally irradiated act-mOVA STAT1–deficient mice compared with STAT1 WT act-mOVA mice (Figure 4, E and F). In summary, these results indicate that the absence of IFN-γR/STAT1 signaling impaired the processing and presentation of exogenous antigen by MHC II in hematopoietic APCs, while promoting the presentation of endogenous self-peptides.

### Effect of IFN-γR/STAT1 signaling on direct and indirect antigen presentation.

**Figure 5:**
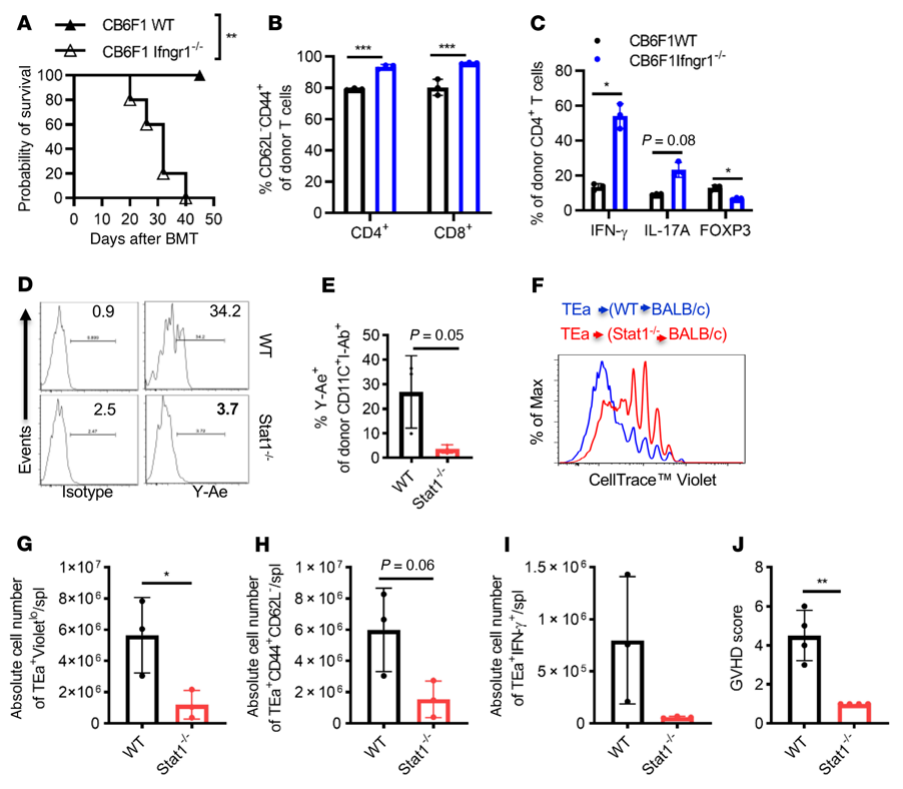
APCs with deficient IFN-γR/STAT1 signaling exhibit increased direct and compromised indirect antigen presentation. (**A**) GVHD was induced in the P→F1 [B6.SJL(H2^b^)→CB6F1 (H2^bxd^)] mouse model using WT or *Ifngr1^–/–^* recipients, and GVHD-induced mortality was monitored. *Ifngr1^–/–^* F1 recipient mice exhibited significantly increased GVHD-induced mortality (MST of 32 days vs. not reached, log-rank test **P < 0.01, *n* = 5 mice/group). (**B** and **C**) Splenic T cell activation was determined by the percentage of CD62L^–^CD44^+^ donor T cells, and differentiation (Th1, Th17, and Treg) was assessed by IFN-γ, IL-17A, and FOXP3 expression in donor CD4^+^T cells on day 7 after BMT. *n* = 3 mice/group. (**D** and **E)** Donor-derived CD11c^+^ cells were assessed on day 18 after transplantation for MHC II (I-A^b^) and associated Ea52-68 peptide presentation (Y-Ae expression) following fully MHC-mismatched BMT in BALB/c mice that received 5 × 10^6^ TCD BMCs from either WT B6 or *Stat1^–/–^* mice after 8 Gy irradiation (*n* = 3 mice/group). (**F**–**J**) In vivo proliferation of TEa-TCR–Tg T cells specific for Ea52-68 peptide presented by I-A^b^. Three weeks after transplantation, 5 × 10^6^ CellTrace Violet–labeled pan–T cells from TEa-TCR–Tg mice were administered i.v. to B6.WT→BALB/c or B6*.Stat1^–/–^*→BALB/c chimeras. Proliferation was determined by CellTrace Violet dilution (**F** and **G**), activation (**H**), and Th1 differentiation (**I**) of antigen-specific TEa TCR^+^ donor T cells (CD4^+^Va2^+^Vb6^+^) on day 5 after DLI (*n* = 3 mice/group). (**J**) Clinical GVHD scores were recorded on day 7 after DLI of TEa-TCR–Tg T cells (*n* = 4 mice/group). Bar graphs represent the mean ± SEM. *P < 0.05, **P < 0.01, and ***P < 0.001, by 2-way ANOVA with Šidák’s correction (**B** and **C**) and 2-tailed Student’s *t* test (**E** and **G**–**J**).

Next, we studied the role of IFN-γR signaling in a P→F1 [B6.SJL (H2^b^)→CB6F1 (H2^bxd^)] model without host irradiation. Under this scenario, the I-Ab molecule in CB6F1 mice presents multiple endogenous parental BALB/c H-2^d^–derived peptides to alloresponsive parental SPCs. Administration of 2.5 × 10^7^ SPCs from B6 mice to CB6F1 *Ifngr1^–/–^* recipient mice led to significantly increased GVHD-induced mortality (Figure 5A) and GVHD target tissue pathology when studied on day 30 after BMT (Supplemental Figure 9). Analysis of peripheral blood, spleen, and lymph nodes on post-transplantation day 7 demonstrated increased expansion and activation of donor CD4^+^ and CD8^+^ T cells (Figure 5B and data not shown). Furthermore, we observed enhanced Th1 and Th17 differentiation of donor CD4^+^ lymphocytes in IFN-γR–deficient CB6F1 recipients, whereas Treg differentiation was significantly reduced (Figure 5C).

Based on the observed impaired presentation of exogenous OVA (Figure 4, B–D) by STAT1-deficient APCs in vitro and in vivo, we postulated that the absence of IFN-γR/STAT1 signaling in donor APCs would impair host antigen presentation via the indirect pathway. To test this hypothesis, we generated BM chimeric mice by reconstituting lethally irradiated BALB/c mice with B6.*Stat1^+/+^* or B6.*Stat1^–/–^* TCD BM cells. Full donor cell hematopoietic engraftment of recipient BALB/c mice was confirmed on day 18 after BMT (data not shown). In these chimeric animals, the host (BALB/c) tissue–derived Eα52-68 peptide can only be presented by donor APCs, i.e., B6, through the indirect pathway via I-Ab. To this end, we first assessed Y-Ae expression on donor CD11c^+^I-Ab^+^ cells. Figure 5, D and E, show significantly more Y-Ae^+^ CD11c^+^ cells in the WT B6→BALB/c mice than in the B6.*Stat1^–/–^*→BALB/c chimeric mice on day 18 after the first transplant, demonstrating defective indirect Eα52-68 peptide presentation of I-Ab by *Stat1^–/–^* B6 donor APCs. These results were further confirmed by transferring Eα52-68 peptide–specific TEa-TCR–Tg donor CD4^+^ T cells into B6.*Stat1^–/–^*→BALB/c or B6→BALB/c chimeras 3 weeks after the initial transplant. In this model, the transferred TEa-TCR^+^CD4^+^ T cells can only respond to the Eα52-68 peptide presented by I-Ab^+^ hematopoietic APCs. We observed significantly reduced in vivo proliferation (Figure 5, F and G), decreased activation (Figure 5H), and Th1 differentiation (Figure 5I) of TEa-TCR–Tg CD4^+^ T cells in B6.*Stat1^–/–^*→BALB chimeric mice compared with B6.*Stat1^+/+^*→BALB/c chimeras. Accordingly, TEa-TCR–Tg DLI-induced GVHD was reduced in *Stat1^–/–^* chimeric mice (Figure 5J) compared with WT chimeras. In our current model, we could not clearly distinguish whether the reduced activation and proliferation of TEa-TCR CD4^+^ T cells was due to reduced processing and presentation or secondary to lower expression of MHC II, leading to subsequently reduced presentation of Eα52-68 peptide or both.

### Absence of STAT1 leads to dysregulation of antigen processing and master genes controlling MHC II expression.

**Figure 6:**
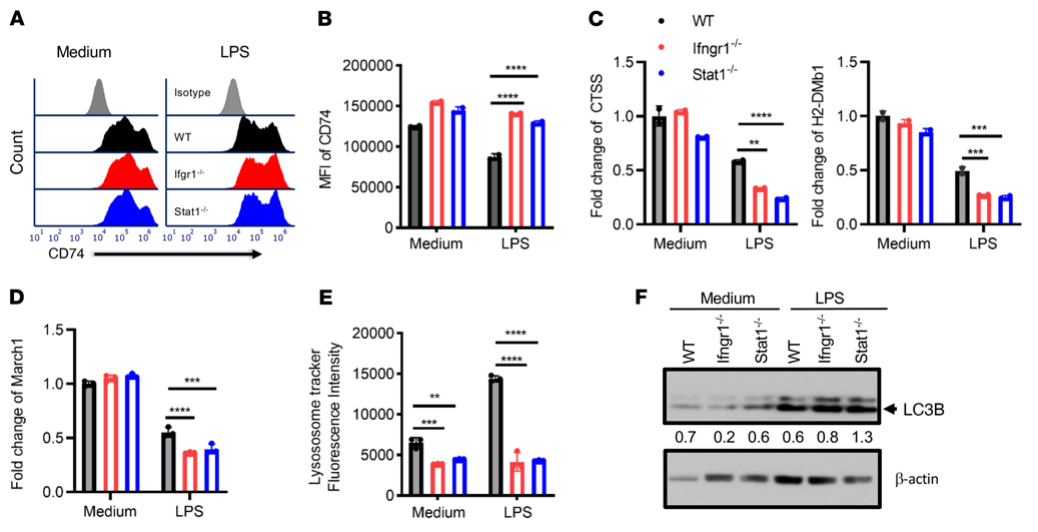
BMDCs with IFN-γR/STAT1 deficiency exhibit impaired peptide exchange and reduced turnover of surface MHC II upon LPS maturation. (**A** and **B**) CD74 expression on CD11c^+^ BMDCs from B6 WT (black), B6.*Ifngr1^–/–^* (red), and *Stat1^–/–^* (blue) mice after LPS maturation for 48 hours (gray histogram represents the isotype control). (**C** and **D**) Real-time quantitative PCR analysis of *Ctss*, *H2-DMb1*, and *March1* mRNA expression in CD11c^+^ BMDCs incubated in the presence or absence of LPS for 4 hours. (**E**) Flow cytometric quantitation of LysoTracker staining of immature or LPS-matured BMDCs from WT, *Ifngr1^–/–^*, or *Stat1^–/–^* mice. (**F**) LC3B expression in WT, *Ifngr1^–/–^* and *Stat1^–/–^* BMDCs after 48 hours of LPS maturation was assessed by Western blotting, semiquantified by ImageJ (NIH). Bar graphs represent the mean ± SEM. **P < 0.01, ***P < 0.001, and ****P < 0.0001, by 2-way ANOVA and Dunnett’s correction for multiple comparisons versus the WT control.

Given the imbalanced antigen presentation of exogenous OVA versus endogenous OVA and the altered MHC II expression levels in IFN-γR/STAT1–deficient mature APCs, we explored potential mechanisms underlying this effect. The transmembrane α- and β-chains of MHC II are assembled in the endoplasmic reticulum, where they associate with the invariant chain (Ii, also known as CD74) (40). The resulting Ii–MHC II complex is transported to a late endosomal compartment termed the MHC II compartment (MIIC), where Ii is digested by cathepsin S (CTSS), leaving only a small fragment called CLIP. This fragment blocks peptide binding until H2-DM interacts with MHC II, which releases CLIP and permits the binding of a specific peptide derived from exogenous proteins degraded in the endosomal pathway. Figure 6, A and B, show that CD74 expression was comparably high in both WT and STAT1*^–/–^* immature BMDCs. As expected, LPS stimulation reduced CD74 expression as BMDCs matured. It was noted that the MFI of CD74 remained higher in both *Ifngr1^–/–^* and *Stat1^–/–^* BMDCs than in WT BMDCs, suggesting a defect in invariant chain release from MHC II. Moreover, CTSS is critical for degrading the Ii chain bound to MHC II before peptide exchange can occur (41). Consistently, the *Ctss* mRNA expression was significantly reduced in both *Ifngr1^–/–^* and *Stat1^–/–^* BMDCs compared with WT BMDCs after LPS stimulation (Figure 6C). Furthermore, as mentioned above, H2-DM is critical in facilitating peptide exchange and displacing the Ii chain with endosome-derived peptides (42, 43). After 4 hours of LPS stimulation, H2-DMb1 mRNA expression levels were significantly lower in *Ifngr1^–/–^* and *Stat1^–/–^* BMDCs compared with levels in their WT counterparts (Figure 6C). These data suggest that peptide exchange in the MIIC may be defective in *Ifngr1^–/–^* and *Stat1^–/–^* BMDCs. Furthermore, posttranslational regulation of MHC II expression by ubiquitination is another critical process in controlling antigen presentation. Usually, the ubiquitination process is suppressed in LPS-matured DCs (44, 45). MARCH1, an E3 ubiquitin ligase constitutively expressed by resting B cells and immature DCs, mediates the ubiquitination of internalized peptide–MHC II complexes at the plasma membrane and in early endosomes targeting these complexes for lysosomal degradation (40, 45, 46). Under normal conditions, the maturation of DCs rapidly terminates MARCH1 expression to spare p–MHC II complexes from lysosomal degradation (45). *March1* mRNA expression (Figure 6D) decreased after LPS maturation, and there was a further reduction in both *Ifngr1^–/–^* and *Stat1^Poison^* BMDCs compared with WT BMDCs upon LPS maturation. These data indicate reduced p–MHC II complex degradation and turnover in APCs with IFN-γR/STAT1 deficiency. In addition to CTSS, many other lysosomal enzymes are critical for the degradation of phagocytosed proteins (47). We observed slightly reduced LysoTracker staining of IFN-γR/STAT1–deficient APCs at baseline in immature BMDCs. However, upon LPS maturation, STAT1- or IFN-γR–deficient APCs failed to show any increase in LysoTracker staining (Figure 6E) compared with WT BMDCs, suggesting that impaired lysosomal activity in STAT1- or IFN-γR–deficient APCs may mitigate the degradation of phagocytosed proteins. As previously reported by others, autophagy delivers cytoplasmic constituents to the autophagosome (48). Furthermore, it plays a critical role in MHC II antigen presentation for cytoplasmic constituents and self-peptides (48). The defective lysosome function in IFN-γR– or STAT-deficient BMDCs prompted us to assess autophagy markers. We measured LC3B expression in *Ifngr1^–/–^* and *Stat1^–/–^* BMDCs by Western blotting, which suggested increased autophagy (Figure 6F). In summary, these data indicate that an absence of STAT1 signaling leads to attenuated lysosomal degradation, defective peptide exchange, and thus impaired exogenous antigen presentation and also provide evidence for increased autophagic activity that might account for the increased endogenous antigen presentation through MHC II. These descriptive results indicate that the absence of IFN-γR/STAT1 signaling in DCs affects multiple antigenic peptide-processing and presentation levels.

## Discussion

In the present study, we have made several observations that may further elucidate how IFN-γR/STAT1 signaling regulates the development of GVHD through host-versus-donor hematopoietic APCs (Supplemental Figure 10). Our main finding suggests that the absence of IFN-γR or STAT1 signaling in recipient hematopoietic APCs promotes GVHD by enhancing the stimulatory capacity of host APCs. We believe our previously published (17) and current results provide the following framework of the role of IFN-γ in GVHD: (a) IFN-γR/STAT1 signaling differentially controls GVHD depending on the target cell. (b) IFN-γR/STAT1 signaling promotes GVHD by inhibiting donor Tregs, while promoting Th1-type responses. (c) Lack of IFN-γR/STAT1 signaling in recipient APCs results in pronounced activation of donor lymphocytes by the enhanced direct allostimulatory capacity of APCs. (d) IFN-γR/STAT1 signaling in donor-derived APCs assists the indirect presentation of host alloantigen to donor T cells to promote GVHD. (e) IFN-γR signaling in hematopoietic APCs functions as a molecular switch in balancing exogenous versus endogenous antigen presentation.

Studies from several investigators support our main observation that the IFN-γR/STAT1 axis has an inhibitory role in hematopoietic APCs. Thus, Thome et al. reported that STAT1 was required for the function of tolerogenic DCs using an EAE mode (49). Similarly, Vogel et al. (50) recently demonstrated that JAK1 inhibitor filgotinib-treated and JAK1-deficient APCs had an enhanced stimulatory capacity also in an EAE model. Interestingly, treatment with filgotinib, a selective JAK1 inhibitor, promoted the expression of MHC II and costimulatory molecules on APCs. In contrast, the absence of JAK1 did not have this effect despite promoting the stimulatory function. Furthermore, Vogel et al. showed that DC-intrinsic IFN-γ–dependent JAK1/STAT1 signaling promotes the expression of PD-L1, leading to enhanced Treg production and peripheral tolerization in the EAE model. Although we also observed IFN-γR/STAT1–dependent regulation of PD-L1 expression on hematopoietic APCs, we did not observe changes in Tregs in our recipients, arguing against a direct Treg-dependent effect in our model.

Our results further contribute to the existing evidence demonstrating differential regulation of GVHD (8–10) by IFN-γ. *Ifngr1^–/–^* or *Stat1^–/–^* donor T cells are severely impaired in their ability to induce GVHD across major and minor histocompatibility disparities, with a concomitant reduction in Th1 differentiation and increased Treg generation, suggesting that the IFN-γ signal pathway in donor lymphocytes is critical in CD4- and CD8-mediated GVHD (14, 15, 51, 52). Neutralization of IFN-γ or administration of IFN-γ–deficient donor T cells in the B6 to B6D2F1 model under nonirradiated conditions resulted in delayed GVHD-induced mortality that was associated with impaired cytotoxic T lymphocyte (CTL) function, reduced elimination of host cells, enhanced Th2 differentiation, and chronic GVHD-like features when compared with recipients of WT grafts (53). In contrast, administration of recombinant IFN-γ protected recipient mice from GVHD and was associated with reduced donor T cell activation (54).

Several studies have reported that the absence of IFN-γR in recipient mice accelerates and enhances GVHD-induced mortality and is associated with severe lung damage (14) and BM failure (25). Burman et al. (14) demonstrated that the detrimental effects of IFN-γ on GVHD induction are mediated through donor T cells, while the protective effects of IFN-γ are mediated through host tissue. In another minor histocompatibility antigen–mismatched (mHA-mismatched), MHC-matched BMT study using *Ifngr1^–/–^* mice as recipients, BM failure occurred as a result of the exposure of donor hematopoietic cells to excessive amounts of IFN-γ accumulation in *Ifngr1^–/–^* recipients (25). Furthermore, Takashima et al. reported attenuated apoptosis in intestinal epithelial cells and crypt stem cells in the absence of IFN-γ signaling (13). Additional studies indicate a complex regulation of the signaling molecules that regulate IFN signaling and the Th1 response during GVHD. The absence of miR-146 in recipients promoted GVHD, which was associated with increased JAK/STAT signaling and involved the regulation of MHC II (55). One study reported that miR-146 acts as a negative feedback controller of LPS/TLR-4 signaling (56). These findings suggest that miR-146 deficiency leads to a much broader inflammatory response than does the activation of STAT1 alone. Paradoxically, deficiency of T-bet in recipient mice attenuated GVHD in MHC-mismatched models (57). This paradoxical role of T-bet and IFN-γR/STAT1 has also been observed in other disease models (12, 58). Interestingly, we detected increased levels of IL-12p70 in our STAT1-deficient recipients on post-BMT day 3 (Supplemental Figure 2). In contrast, recipient mice with T-bet deficiency had reduced IL-12 and IFN-γ production (57, 59). Furthermore, mice with T-bet–deficient APCs are impaired in their ability to induce Th1 differentiation and antigen-specific T cell activation (60), which may explain the differential GVHD induction in IFN-γR/STAT1– versus T-bet–deficient recipient mice.

Our results support the notion that IFN-γR/STAT1 signaling in host hematopoietic APCs may be an early negative regulator of host endogenous antigen presentation and MHC II expression and thus suppress the activation of donor lymphocytes. We postulate that IFN-γ suppresses host-derived peptide–MHC II complex presentation on host hematopoietic APCs at early time points after transplantation, leading to reduced donor TCR engagement. Our findings are in potential discord with the results by Delisle, who showed reduced MHC II expression on day 8 following induction of mHA-mismatched GVHD (25). There are several possible reasons for these apparent discrepancies, with the most crucial being that Delisle et al. studied MHC II expression in GVHD target organs on nonhematopoietic cells and not in BMDCs. We made preliminary observations that MHC II expression was reduced in epithelial cells of GVHD organs (e.g., intestinal epithelial cells, data not shown). Furthermore, based on our studies, the time point of studying MHC II expression will be crucial. We argue that the priming of donor lymphocyte activation should occur before days 3–5 after BMT and that hematopoietic APCs will be quickly eliminated, given the intense lymphohematopoietic graft-versus-host (LHGVH) response of activated donor T cells.

Notably, our data demonstrated that IFN-γR/STAT1 signaling in APCs had differential effects on GVHD development depending on whether the APCs originated from the host or the donor. An absence of IFN-γR/STAT1 signaling promoted direct antigen presentation on host APCs and therefore led to increased GVHD. Conversely, the lack of IFN-γR/STAT1 signaling mitigated GVHD by reducing the indirect presentation of host antigens by donor APCs to donor T cells. Capitini and colleagues reported that in fully chimeric mice generated with STAT1^–/–^ BM, GVHD induction is prevented by delayed allogeneic WT donor lymphocyte infusion. Their explanation for this attenuated GVHD was that STAT1-deficient CD9^–^SiglecH^hi^ donor plasmacytoid DCs (pDCs) were tolerogenic and crucial for the reduced induction of GVHD (61) through increased IL-10 and reduced IL-12 and IFN-α production. Using fully chimeric mice generated with *Ifngr1^–/–^* BM, we observed reduced GVHD by delayed allogeneic WT donor lymphocyte infusion. As stated above, our results suggest that impaired indirect host antigen presentation may be an additional mechanism of the GVHD-mitigating effects of IFN-γR/STAT1–deficient APCs, resulting in attenuated activation of host peptide–specific donor T lymphocytes and, consequently, suppressed GVHD induction.

Nonselective JAK inhibitors like Rux have recently been approved for steroid-refractory GVHD. We posit that the Rux-dependent anti-GVHD effects are not only mediated by direct inhibition of donor T cell responses and IFN-γ–mediated cytopathic effects in the GVHD target tissue but also involve mitigation of indirect presentation of host antigens by donor APCs. However, we would caution that the presence of host APC JAK inhibitors may worsen the development of GVHD. Our data provide evidence that defective IFN-γ/STAT1 signaling may increase endogenous antigen presentation with the potential for enhancing autoimmunity and impaired protective immunity against exogenous pathogens. Consistent with this implication, Shao et al. (62) recently reported that STAT1-deficient hosts are more susceptible to the induction of chronic GVHD with enhanced anti-dsDNA autoantibody responses, increased proteinuria, and mortality. Furthermore, our results may allow us to test whether engineering the IFN-γR/JAK1/STAT1 signaling pathway in APCs could be of value in achieving a more efficient presentation of endogenous tumor–associated peptides to CD4^+^ T cells and potentially enhancing tumor responses. In summary, our data document a regulatory role of IFN-γ/STAT1 signaling in hematopoietic APCs that modulates GVHD induction by suppressing direct antigen presentation of host APCs while promoting indirect antigen presentation through donor APCs.

## Methods

### Mice.

WT 129S6/SvEv mice [129*.Stat1^+/+^*(H2^b^)], C57BL/6 [(H2^b^), B6], B6.SJL-Ptprca (B6.SJL, H2^b^), BALB/c (H2^d^), CB6F1, B6.Act-mOVA, B6.IFN-γ receptor 1 (B6.*Ifngr1^–/–^*), BALB/cByJ.*Ifngr1^–/–^*, B6.*Stat1^–/–^*, OTII, C.FVB-Tg (CAG-Luc-GFP, BALB/c-Luc), and B6.TEa-TCR–Tg mice (B6.TEa) were purchased from The Jackson Laboratory or Taconic. Act-mOVA-*Stat1^–/–^* (OVA-*Stat1^–/–^)* and Act-mOVA-*Ifngr1^–/–^* (OVA-*Ifngr1^–/–^*) mice were created by breeding B6.Act-mOVA mice with B6.*Stat1^–/–^* and B6.*Ifngr1^–/–^* mice, respectively. CB6F1.*Ifngr1^–/–^* mice were generated by breeding B6.*Ifngr1^–/–^* mice with BALB/cByJ.*Ifngr1^–/–^* mice. B6.*Stat1^Poison^* (*Stat1^Poison^*) mice (ENU mutagenesis–derived mutant with complete loss of STAT1 function) were generated by K. Hoebe (Cincinnati Children’s Hospital Medical Center) (30). All mice were used between 8 and 12 weeks of age, housed in an autoclaved microisolator environment, and provided with sterile water and irradiated food ad libitum. All manipulations were performed in a laminar flow hood.

### BMT and induction of GVHD.

Mice underwent BMT as described previously (51). Mice received total body irradiation (TBI) as follows: mice on the B6 background received 1,075 cGy; mice on the 129-SvEv background received 1,044 cGy; and BALB/c mice received 800 cGy TBI. Lethally irradiated mice were reconstituted with 5 × 10^6^ TCD allogeneic or syngeneic BMCs. TCD was performed using CD90.2 microbeads (Miltenyi Biotec). GVHD was induced by coinjection of allogeneic splenic cells or selected T cell populations, as described below. For the B6 →CB6F1 (P→F1) graft-versus-host reaction (GVHR) model, nonirradiated F1 recipient mice (WT or *Ifngr1^–/–^*) received 5 × 10^6^ BMCs and 25 × 10^6^ SPCs from B6.SJL mice or 3 × 10^6^ TEa cells. Pan–T cells or naive CD4^+^ T cells from SPCs were purified using the Pan T Isolation Kit II or the CD4^+^ Isolation Kit (Miltenyi Biotec) according to the manufacturer’s recommendations (purity ≥95%), and in some experiments, they were labeled with 5 μM CFSE (Invitrogen, Thermo Fisher Scientific) to assess in vitro and in vivo proliferation, according to the manufacturer’s instructions. For in vivo OVA pulse experiments, mice were i.p. injected with 100 μg/mouse OVA (Ovalbumin EndoFit, InvivoGen) emulsified in incomplete Freund adjuvant (IFA) (InvivoGen) 1 hour before irradiation. For survival experiments, the degree of systemic GVHD was measured with a validated clinic scoring system (51). To avoid bias from cage-related effects, animals in different groups were randomized between cages.

### Creation of BM radiation chimeras and DLI.

For 129SvEv-background *Stat1^–/–^* chimeras (*Stat1^–/–^→Stat1^–/–^*), lethally irradiated 129*.Stat1^+/+^* mice were infused with 5 × 10^6^ TCD BMCs from either 129.*Stat1^–/–^* or 129.*Stat1^+/+^* donors. The absence of STAT1 in host hematopoietic cells was confirmed by testing intracellular STAT1 expression in PBMCs 2 months after BMT (data not shown). For B6.*Stat1^Poison^* chimeras (B6.SJL^–^→B6.*Stat1^Poison^*), in which STAT1 deficiency was confined to the nonhematopoietic organs, lethally irradiated B6.*Stat1^Poison^* or B6 WT mice received 5 × 10^6^ TCD BMCs from B6.SJL mice. For B6.*Ifngr1^–/–^* to WT chimeras (B6.*Ifngr1^–/–^*→B6.SJL), in which IFN-γR1 deficiency was confined to hematopoietic cells, lethally irradiated B6.SJL WT mice received 5 × 10^6^ TCD BMCs from B6.*Ifngr1^–/–^* mice (B6.*Ifngr1^–/–^*→B6.SJL). To create B6.SJL→B6 *Ifngr1^–/–^* chimeras in which IFN-γR deficiency was confined to the nonhematopoietic organs, lethally irradiated B6.*Ifngr1^–/–^* mice received 5 × 10^6^ TCD BMCs from B6.SJL mice. Subsequently, these mice were reconditioned with TBI and injected with 5 × 10^6^ BMCs and 5 × 10^6^ splenic cells from BALB/c or BALB/c-Luc mice for induction of GVHD. For *Stat1^–/–^* or WT to BALB/c chimeras, lethally irradiated BALB/c mice received *Stat1^–/–^* or WT TCD BMCs. Examination for chimerism was performed on day 18, and GVHD was induced by DLI of 3 × 10^6^ CellTrace Violet–labeled TEa cells on post-BMT day 20.

### BMDC generation and phenotypic and functional assays.

BMDCs were generated from murine BMCs cultured in a medium containing murine GM-CSF (20 ng/mL, Peprotech). On days 6–7, nonadherent cells were harvested, and CD11c^+^ DCs were purified with CD11c^+^ microbeads (purity >90%, Miltenyi Biotec) and matured with LPS (100 ng/mL) for 4–48 hours. For the gene expression assay, cells were harvested 4 hours after LPS, and for the phenotypic assay, cells were harvested 24 hours or 48 hours after LPS maturation. For in vitro antigen presentation experiments, BMDCs were pulsed with OVA 100 ng/mL or OVA 323–339 for 2 hours, followed by LPS stimulation overnight. Suspension cells were fixed with 0.5% paraformaldehyde (PFA) and then cocultured with CFSE-OTII cells at a 1:5 ratio for 4–5 days. For the MLR assay, LPS-matured BMDCs were irradiated with 3,000 cGy and used as stimulator cells, followed by coculturing with alloresponder cells labeled with 5 μM CFSE. Cell proliferation was detected by CFSE dilution using flow cytometry. Alternatively, the proliferation of responder cells was measured by a ^3^H-thymidine incorporation assay (1 μCi/well, 0.037 MBq) during the last 18 hours of culturing, and cells were then harvested and counted (presented as cpm) using a TopCount Microplate (Packard). All experiments were performed in triplicate.

### CRISPR/Cas9-mediated Stat1 genomic targeting.

*Stat1* CRISPR RNA (crRNA) (2.5 nM, Integrated DNA Technologies [IDT], predesigned crRNA) or negative control crRNA (IDT) plus tracrRNA-ATTO (IDT) was incubated together with 0.15 μg/μL TrueCut Cas9 Protein v2 (Invitrogen, Thermo Fisher Scientific, A36498) for 10 minutes at room temperature to form the CRISPR-CAS9-gRNA ribonucleoprotein (RNP) complex. Freshly isolated PBMCs (5 × 10^6^) were transfected with an RNP mixture with a Lipofectamine CRISPRMAX Cas9 Transfection kit (Invitrogen, Thermo Fisher Scientific, CMAX00003) following the kit’s instruction. Twenty-four hours after transfection, the medium was replaced with 1 mL fresh culture medium (10% human AB serum AIM-V) for each well of a 24-well plate. Three days after transfection, the ATTO 550^+^ cells were sorted and irradiated at 3,000 cGy. The irradiated cells were cocultured with 5 μM CFSE-labeled allo-human PBMCs at a 1:1 ratio in 96-well round-bottomed plates. After 4 days of culturing, the cells were harvested for flow cytometric analysis. All experiments were performed in triplicate. The knockdown effects within the PBMC bulk population were confirmed by Western blotting.

### JAK inhibitor Rux treatment.

Human PBMCs were depleted with CD3 and CD56 microbeads (Miltenyi Biotec). CD3-/CD56-depleted PBMCs were treated with 5 μM Rux (Selleck Chemicals) for 4 hours, followed by stimulation with 100 μg/mL LPS for an additional 48 hours. After stimulation, the cells were irradiated with 3,000 cGy. To analyze the allostimulatory capacity, irradiated cells were cocultured with 5 μM CellTrace Violet–labeled allo-human PBMCs at a 1:1 ratio for 4 days; CD25 expression and Violet dilution on CD4^+^ and CD8^+^ T cells were analyzed by flow cytometry.

### Flow cytometric analysis.

Single cells were prepared and analyzed using flow cytometry with an LSR II or Canto II flow cytometer (BD Biosciences) and FlowJo or FCSExpress software. Cells were stained for 30 minutes at 4°C. For intracellular staining, 1 × 10^6^ cells were stimulated with PMA (50 ng/mL) and ionomycin (1 μg/mL) in the presence of monensin (10 μg/mL) in 1 mL complete medium for 4 hours at 37°C in 5% CO_2_. The cells were stained with surface antibodies for 30 minutes at 4°C, fixed and permeabilized, and then subjected to intracellular staining. The antibodies used for these studies are listed in Supplemental Table 1.

### In vivo labeling of mouse cells with BrdU.

SPCs were isolated 24 hours after i.p. injection with 200 μL (2 mg) of a 10 mg/mL BrdU solution (BD Biosciences). Then, SPCs were stained for FITC-labeled anti-BrdU and other markers.

### LysoTracker labeling.

Treated or untreated BMDCs were loaded with 10–75 nM LysoTracker Deep Red (Invitrogen, Thermo Fisher Scientific) according to the manufacturer’s instructions by incubating the cells with dye for 30 minutes at 37°C, followed by washing and analysis by flow cytometry.

### Histology.

For histopathological analysis of GVH target tissues, samples were collected and fixed in 10% formalin. Samples were then embedded in paraffin, cut into 5 μm thick sections, and stained with H&E for histological examination.

### BLI.

T cell expansion was analyzed on the basis of luciferase signal intensity. Briefly, mice were i.p. injected with luciferin (200 ng/g body weight) and euthanized 7 minutes later. The organs were then imaged for 30 seconds using a Xenogen IVIS 100 (PerkinElmer). The imaging data were analyzed with Living Image Software (PerkinElmer) and are presented as photons per second.

### Real-time quantitative PCR analysis.

Total RNA was isolated with TRIzol reagent (Invitrogen, Thermo Fisher Scientific), and cDNA was generated with the Superscript III RT kit (Invitrogen, Thermo Fisher Scientific). PCR was carried out with SYBR Green PCR Master Mix (Applied Biosystems). The following primer sets were used: *Ctss, H2Dmb1,* and *March1* (obtained from QIAGEN) and β-actin sense, 5′-GAA ATCGTGCGTGACATCAAAG-3′ and antisense, 5′-TGTAGTTTCATGGATGCCACAG-3′. The average Ct for each gene was determined from duplicate or triplicate reactions. Expression of the target gene was normalized to β-actin using the difference between the Ct values to generate the ΔCt. To compare the effects of *Ifngr1* or *Stat1* gene knockout and LPS treatment on DCs, WT immature DCs were set as the reference. The other groups were compared to obtain the ΔΔCt value. The fold change of target gene expression was determined using the 2^−ΔΔCt^ formula.

### Western blot.

Proteins were extracted from cultured cells for immunoblotting using a modified RIPA buffer. Total protein lysates (25–40 μg/mL per lane) were separated by 4%–12% PAGE (Bio-Rad). After transfer, the blots were incubated with antibodies against LC3II (Cell Signaling Technology) and β-actin (MilliporeSigma) and then visualized using SuperSignal Chemiluminescent Substrate (Pierce, Thermo Fisher Scientific).

### Statistics.

Survival data are presented as Kaplan-Meier survival curves, and differences between groups were analyzed by log-rank test using GraphPad Prism, version 9.4.1 (GraphPad Software). The Shapiro-Wilk test was used to assess data normality. Differences between group mean values were tested using a 2-tailed Student’s *t* test or a Mann-Whitney *U* test for nonparametric data. A *P* value of less than 0.05 was considered statistically significant. When more than 2 groups were compared, a 2-way ANOVA was used with Dunnett’s correction for multiple comparisons with a control group. Šidák’s correction was used for comparison of groups of means.

### Study approval.

The Columbia University IACUC approved all animal procedures.

## Author contributions

CL and HM contributed equally to this work and share first authorship. They designed and conducted the experiments, generated the manuscript figures, and wrote the manuscript. CL wrote the initial draft of the manuscript and is therefore listed first as co-author. LS, HW, LW, S Li, and KH conducted or assisted with performing experiments and analyzing the data. ARS and S Lagana reviewed the histology. SSP helped with statistical analysis of the data. YGY and S Lentzsch contributed to the design of the experiments, analysis of the data, and writing of the manuscript. MYM designed and supervised the experiments and wrote the manuscript.

## Supplementary Material

Supplemental data

## Version 1 11/29/2022

In-Press Preview

## Version 2 02/01/2023

Electronic publication
