# Supplementary material for: IFN-γR/STAT1 signaling in recipient hematopoietic antigen-presenting cells suppresses graft-versus-host disease
Source: J Clin Invest. 2023 Feb 1;133(3):e125986. doi: 10.1172/JCI125986 (PMC9888368; doi:10.1172/JCI125986)

Supplemental Material for

IFNGR/STAT1 Signaling in Recipient Hematopoietic Antigen Presenting Cells Suppresses Graft versus Host Disease

Caisheng Lu[1+], Huihui Ma[1+], Liangsong Song[1], Hui Wang[1], Lily Wang[1], Shirong Li[2], Stephen Lagana[3], Antonia R. Sepulveda[3], Kasper Hoebe[4,5], Samuel S. Pan[6], Yong-Guang Yang[1], Suzanne Lentzsch[2], and Markus Y. Mapara*[1, 2]

[1]Columbia Center for Translational Immunology. Columbia University, New York City, NY.
[2]Division of Hematology-Oncology, Columbia University, New York City, NY
[3]Department of Pathology, George Washington University, Washington, DC
[4]Department of Pediatrics, University of Cincinnati, OH
[5]Janssen Research & Development, Spring House, PA
[6]Herbert Irving Columbia Cancer Center, Cancer Biostatistics Shared Resource, New York, NY

* To whom correspondence should be addressed:
Markus Y. Mapara, MD, PhD
Department of Medicine,
Division of Hematology-Oncology
Columbia Center for Translational Immunology
Columbia University, College of Physicians and Surgeons
Harkness Pavilion 11-31
New York, NY 10032
PHONE: 646-317-5689
email: mym2111@columbia.edu

1. Supplemental Methods

Supplemental Table I Antibodies

Marker	Flurochrome	Clone	Manufacturer	Catalog #	Dilution
**CD4**	PE-CY5	H129.19	BD Biosciences	553654	1:500
**CD8a**	Alexa Fluor700	53-6.7	BD Biosciences	557959	1:500
**CD62L**	PE	MEL-14	Biolegend	104408	1:500
**CD44**	APC-CY7	IM7	Biolegend	103028	1:500
**CD11b**	PE-CY7	M1/70	BD Biosciences	552850	1:500
**CD11c**	Percpcy5.5	HL3	BD Biosciences	560584	1:100
**B220**	APC-CY7	RA3-6B2	BD Biosciences	552094	1:500
**H-2K^b**	BV421	AF6-88.5	BD Biosciences	562942	1:100
**H-2D^d**	BV786	34-2-12	BD Biosciences	742465	1:100
**I-Ab**	FITC/PE	AF6-120.1	BD Biosciences	553551/553552	1:500
**CD86**	PE	GL1	BD Biosciences	553692	1:100
**PD-L1**	APC	MIH5	BD Biosciences	564715	1:500
**CD74**	FITC	In-1	BD Biosciences	561941	1:500
**IFN-g**	PE	XMG1.2	BD Biosciences	554412	1:100
**CD25**	PE-CY7	PC61	BD Biosciences	552880	1:200
**Y-Ae**	FITC	ebio-YAe	eBioscience	11-5741-82	1:100
**IL-17A**	Alexa Fluor488	TC11-18H10	BD Biosciences	560220	1:100
**FOXP3**	Alex Fluor488	FJK-16s	eBioscience	53-5773-82	1:100

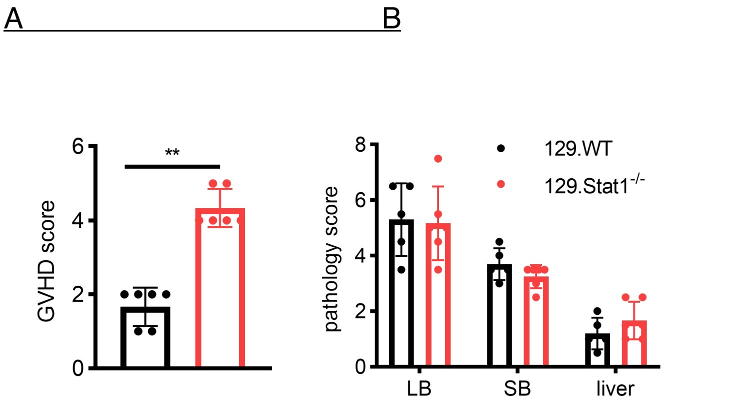

Suppl. Fig 1. Exacerbated morbidity measured by GVHD score in STAT1 deficient host. A) Increased GVHD scores day+4 post-BMT are shown in 129.Stat1$^{-/-}$ host receiving 5x10^{6} BMC and 10x10^{6} splenocytes from BALB/c mice. Mann-Whitney U test ** $p<0.01$. **B)** Histopathological scores in target organs, including large bowel (LB), small bowel (SB), and liver, was shown. Two independent experiments were combined.

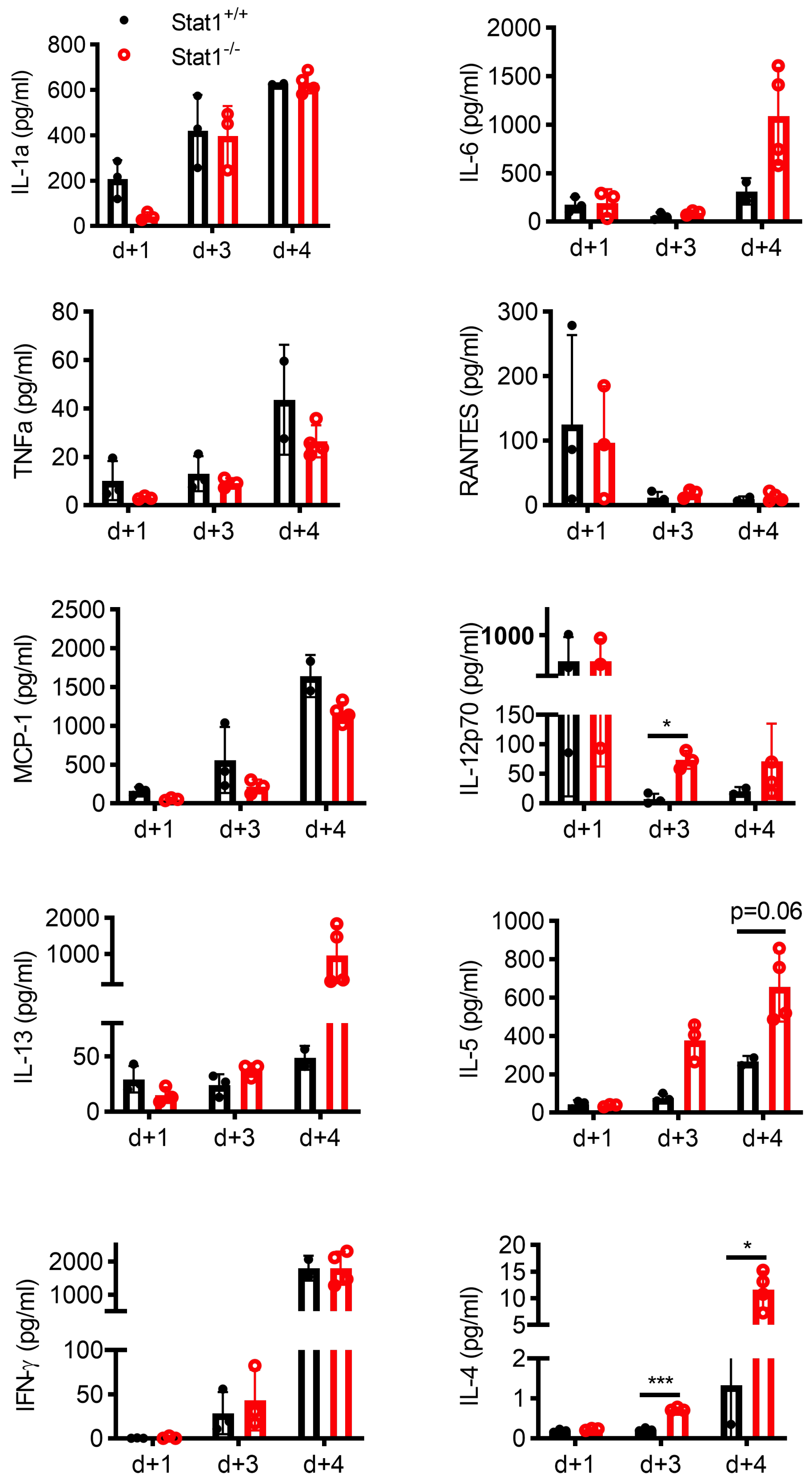

Suppl. Fig 2. Inflammatory cytokine profiles in Stat1$^{-/-}$ recipient mice in comparison with wild-type recipient mice.

GVHD was induced in the fully MHC-mismatched (BALB/c [H2^d] to 129Sv [H2^b]) strain combination. Lethally irradiated (1,044 cGy) 129.Stat1$^{-/-}$ or 129.Stat1$^{+/+}$ mice received 5x10^6 BMC and 1x10^7 splenocytes from BALB/c mice. Serum cytokine profiles were studied on day+1, +3, and d+4 post-BMT with 2–4 mice/group at each time point. Serum levels of individual animals are shown. Horizontal bars denote the mean cytokine serum concentration of the group. Bar graphs represent the mean ± SEM, p values were calculated 2-way ANOVA and Sidak correction for multiple comparisons * $p<0.05$, ** $p<0.01$, *** $p<0.001$.

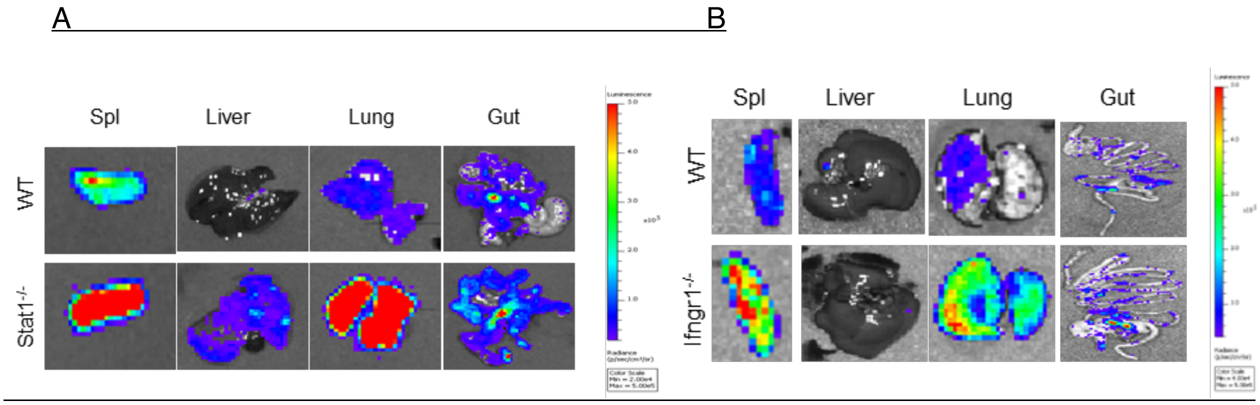

Suppl. Fig 3. Increased alloreactive donor lymphocyte expansion and infiltration in recipient mice with IFNGR or STAT1 deficiency.

A) BLI analysis of the *in vivo* expansion of alloreactive donor BALB/c-luc T cells and target organ infiltration (spleen, liver, lung, and gut) in B6 (WT) or B6.Stat1$^{-/-}$ recipients on day+6 post-BMT. Representative results of one of 3 independent experiments with 3-4 animals per group are shown. **B)** Fully MHC-mismatched GVHD induction following lethal irradiation in B6 (WT) vs. B6.Ifngr$^{-/-}$ recipients using $1x10^7$ splenocytes and $5x10^6$ BMCs from BALB/c-luc mice. Infiltration and expansion of BALB/c-luc lymphocytes in the organs of recipient animals were monitored on day +7 post-BMT using BLI. Representative results of bar graphs in Fig.1D and Fig.1H.

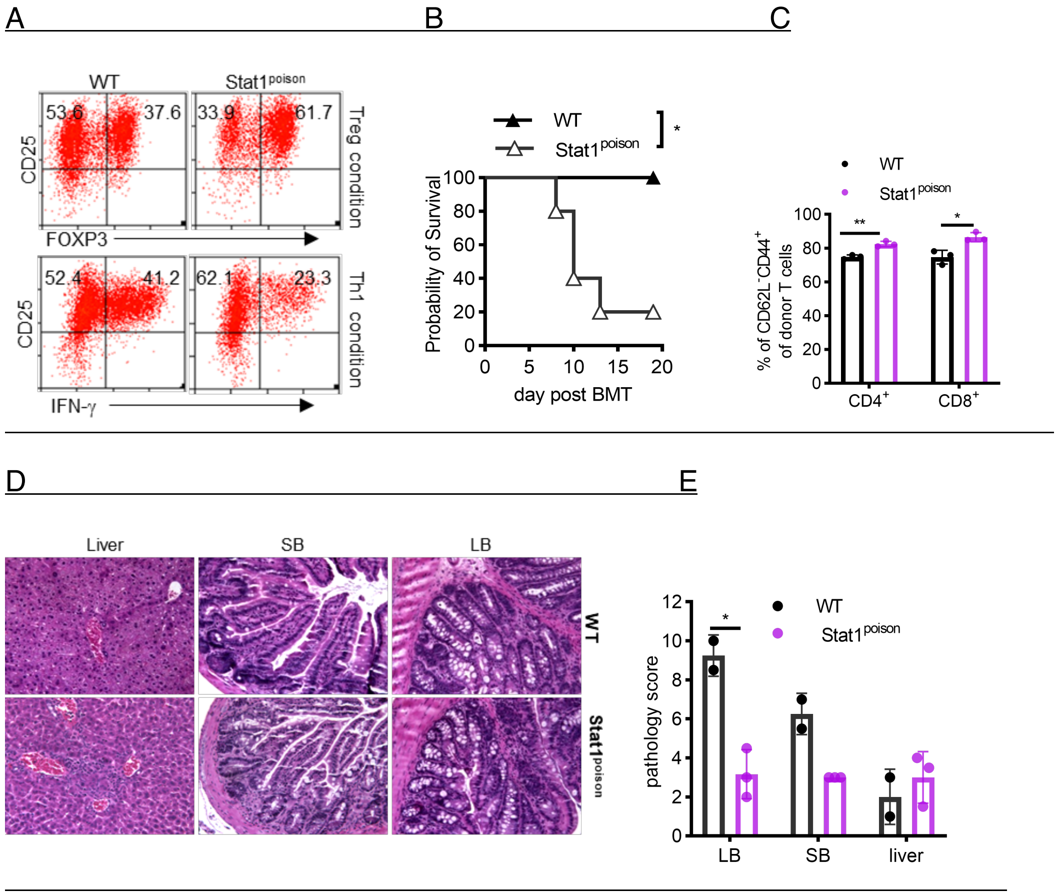

Suppl. Fig 4. Characterization of B6.Stat1^{Poison} mice

A) Enhanced T_{reg} development and reduced Th1 differentiation in B6.Stat1^{poison} mice. CD4 T cells from wild-type B6 (B6.SJL) or B6.Stat1^{poison} mice were assessed for CD25 expression and Foxp3 or IFN-γ intracellular staining following three days of culture under Th1 or T_{reg} conditions. **B)** GVHD was induced in the fully MHC-mismatched (BALB/c [H2^d] to B6 [H2^b]) strain combination. Wild-type B6 or B6.Stat1^{poison} mice were lethally irradiated with 1,075rad and received $3x10^6$ BALB/c BMC and $3.0x10^6$ splenocytes. Survival analysis following induction of GVHD (Median survival time (MST) not reached vs. 11 days, log-rank test p=0.014) in wildtype compared to B6.STAT1^{poison} mice. Representative data are from 3 similar experiments with 5 animals per group. **C)** On day +8 post-BMT, animals were euthanized, and splenocytes were analyzed by flow cytometry for the activation status of donor-derived $CD4^+$ and $CD8^+$ cells based on $CD62L^-CD44^+$. Representative results of 3 independent experiments are shown with 5 mice per group. **D, E)** GVHD target organs were isolated on day+8 post-BMT. Histology with HE staining (100x) and pathology scores of the liver, small bowel (SB), and large bowel (LB) are shown. Bar graphs represent the mean ± SEM, p values were calculated by 2-way ANOVA with Sidak correction for multiple comparisons * p<0.05, ** p<0.01.

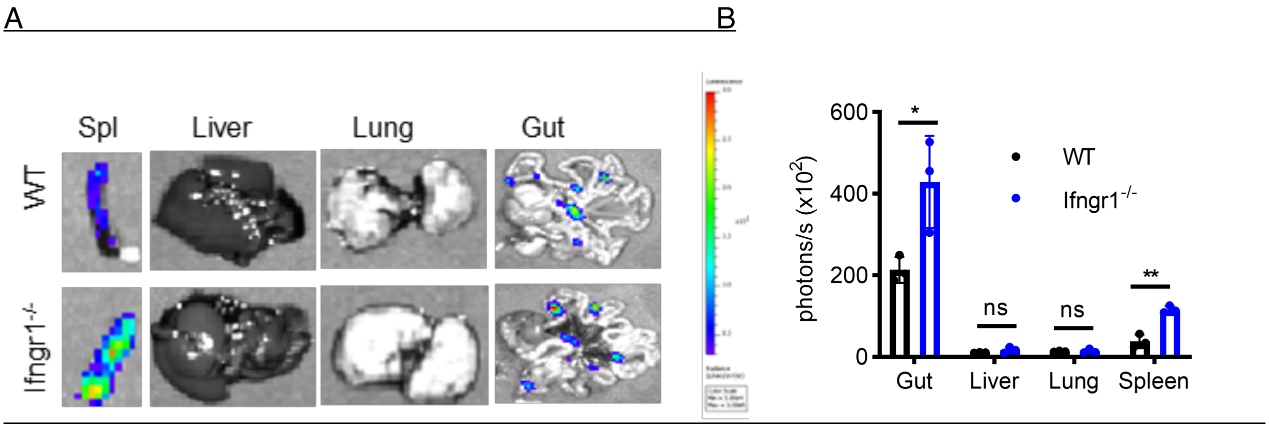

Suppl. Fig 5. Donor lymphocyte expansion in target organs in recipient mice with IFNGR deficiency in comparison to wild-type recipients on day+4 post -BMT. Fully MHC-mismatched GVHD induction following lethal irradiation in WT vs. B6.Ifngr$^{-/-}$ recipients using 1x10^7 splenocytes from BALB/c-luc mice. A, B) Infiltration and expansion of BALB/c-luc T lymphocytes in recipient animals' spleen, liver, lung, and gut were monitored on day+4 post-BMT using BLI (n=3 mice/group). A summary bar graph shows representative results of one of 2 independent experiments with 3 animals/group. Bar graphs represent the mean ± SEM, p values were calculated by 2-way ANOVA with Sidak correction for multiple comparisons * $p<0.05$, ** $p<0.01$.

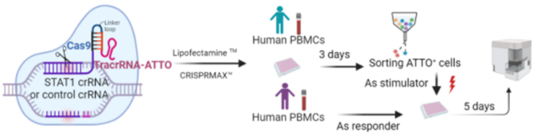

A

B

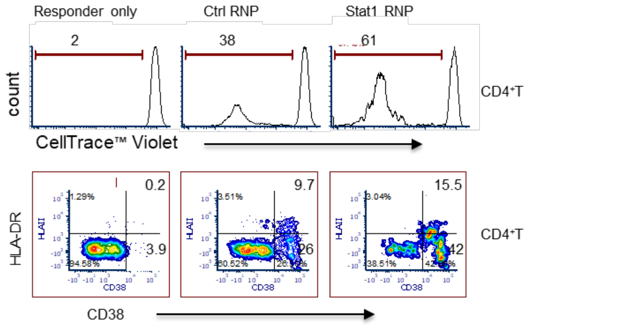

Suppl. Fig 6. Knockdown of Stat1 in human PBMCs by CRISPR/Cas9 enhances their allo-stimulatory capacities.

A) Schematic of experimental procedure. **B)** Allo-CD4+ T cell proliferation and activation were detected by flow cytometry after 4 days of coculturing of stimulator and responder cells with a ratio of 1:1. Representative result of 2 independent experiments with two different donors.

A

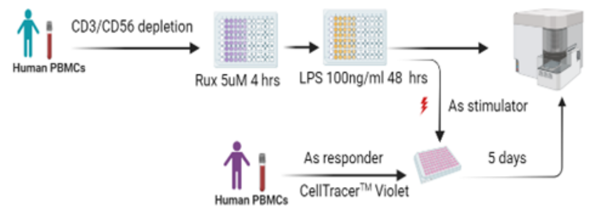

B

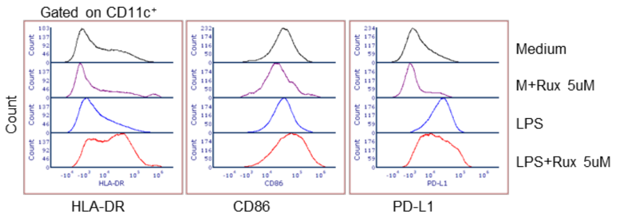

C

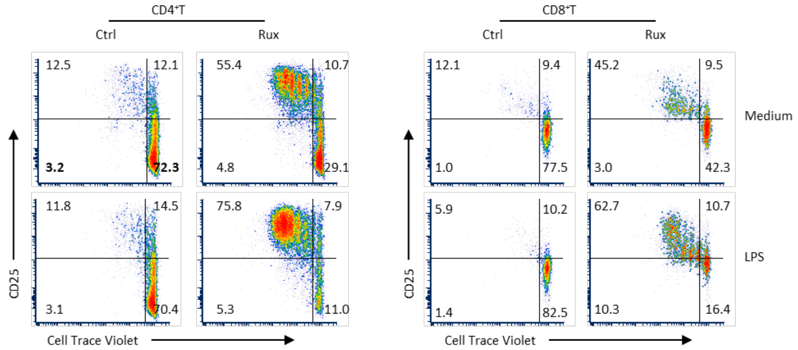

Suppl. Fig 7. JAK inhibitor Ruxolitinib (Rux) treated hPBMCs have increased HLA II, CD86 and reduced PD-L1 expression on CD11C+ cells, and enhanced allo-stimulatory capacity. A) Experimental design. B) Phenotype of Rux (5uM) treated human PBMCs 48hrs after LPS stimulation. **C**) Allo-human PBMCs labeled with CellTrace-Violet were cocultured with Rux and LPS (100ng/ml) treated cells for 4 days, and CD25 expression and Violet dilution on CD4+ and CD8+ T cells were studied by FACS. Representative of 2 independent experiments.

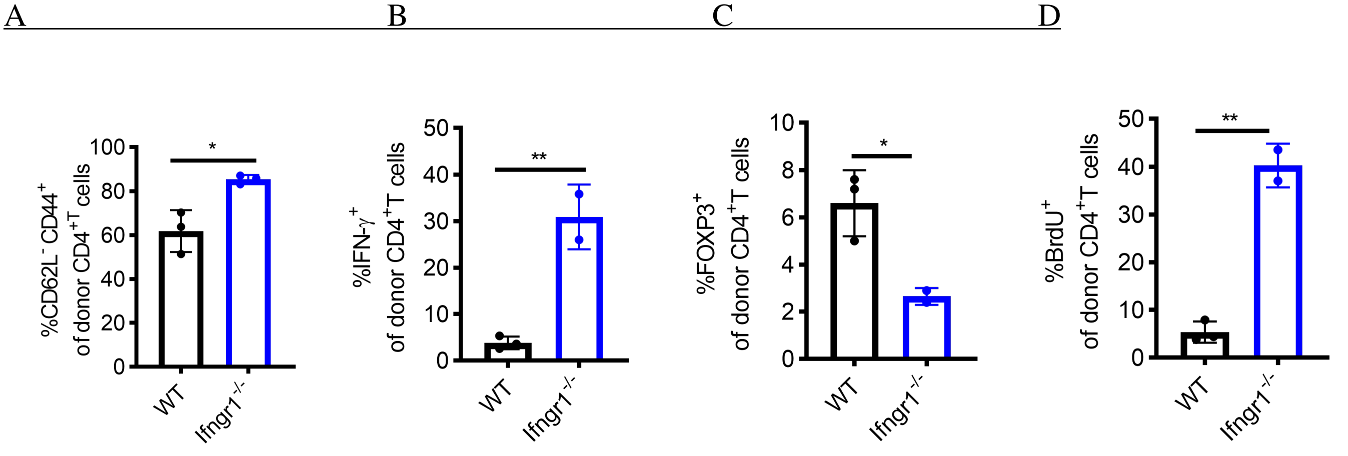

Suppl. Fig 8. Enhanced activation of TEa-donor T cells in CB6F1 recipient mice with IFNGR-deficiency. GVHD was induced in the B6 to CB6F1 mouse model. TEa splenocytes ($1x10^7$) recognizing Ea52-68 peptide presented by I-A^b were i.v. injected into WT or $Ifngr^{-/-}$ CB6F1 mice. Thereafter, mice were injected i.p. with BrdU. On day +7, the mice were euthanized. Splenocytes were harvested and assessed for T cell activation ($CD62L^-$and CD44 expression) in donor CD4 and CD8 T cells (**A**)**,** Th1(**B**), T_{reg} (**C**) differentiation by staining for IFN-γ and FOXP3 in CD4 T cells and $CD4^+$donor T cell proliferation (**D**) by BrdU incorporation. Bar graphs represent the mean ± SEM, p values were calculated by 2-tailed t test * p<0.05, ** p<0.01.

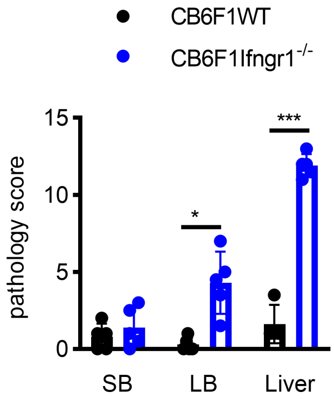

Suppl. Fig 9. Enhanced inflammation in GVHD target tissues in CB6F1 recipient mice with IFNGR deficiency.

GVHD was induced in the P →F1 (B6.SJL[$H2^b$] →CB6F1 [$H2^{bxd}$]) mouse model using wild-type or $Ifgnr^{-/-}$ recipients receiving $5x10^6$ BMCs and $2.5x10^7$ splenocytes from B6 mice without irradiation. GVHD target organs were isolated on day +30 post-BMT. Pathology scores of the small intestine (SB), colon (LB), and liver are shown. Representative of 2 independent experiments. Bar graphs represent the mean ± SEM, p values were calculated by 2-way ANOVA with Sidak correction for multiple comparisons * p<0.05, ** p<0.01, *** p<0.001.

Suppl. Fig. 10. Defective IFNGR/STAT1 signaling leads to enhanced endogenous but compromised exogenous Ag presentation (Created with BioRender.com)

A. Enhanced direct endogenous Ag presentation by the recipient APCs with defective IFNGR/STAT1 signaling in the BALB/→B6 MHC-mismatched BMT setting (left) and in the haplotype MHC-mismatched B6 to CB6F1 BMT model (right).

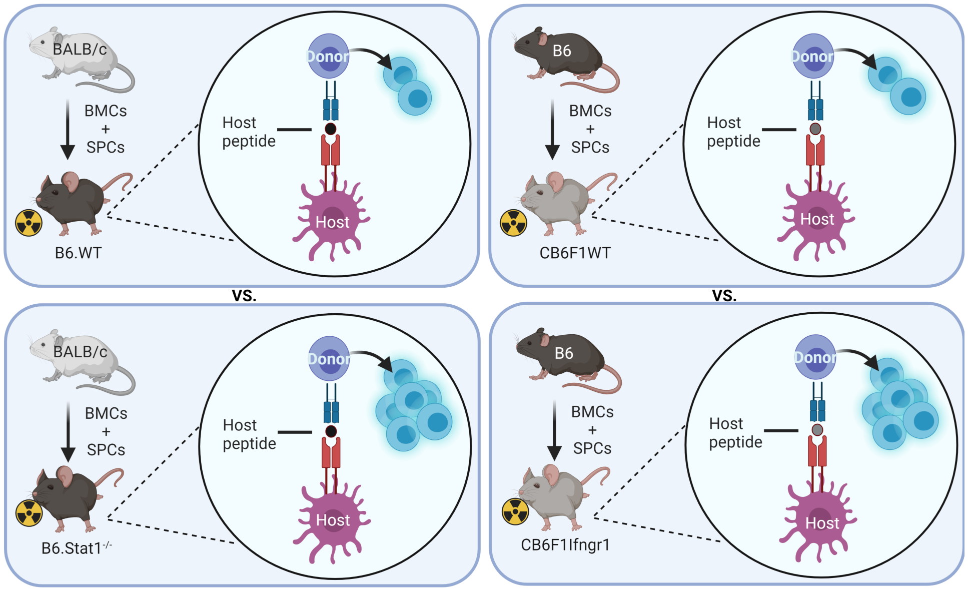

B. Compromised indirect host Ag presentation by the donor-derived APCs with defective STAT1 signaling (B6 →BALB/c) chimeric mice as recipients. Proliferation and activation of the adoptively transferred TEa lymphocytes were used as readouts of the indirect Ag presentation.

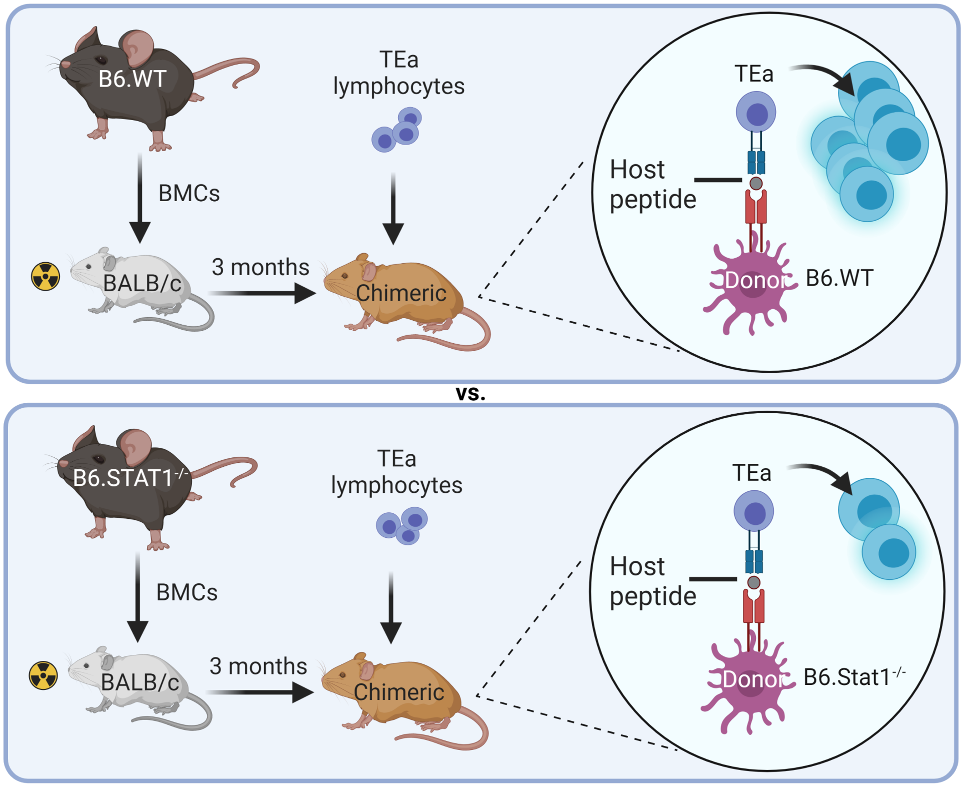

C. <u>Presentation of exogenous OVA protein was compromised in Stat1$^{-/-}$ B6 mice (left); in contrast, constitutively expressed endogenous ovalbumin Ag presentation was enhanced in the Stat1$^{-/-}$ host (right).</u>

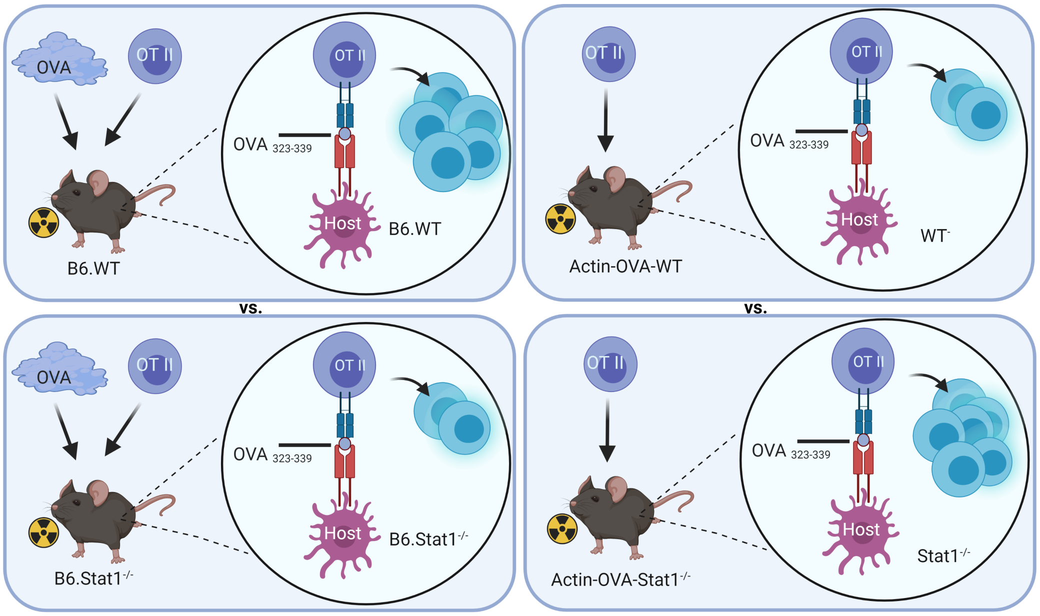

Supplement: Supplemental data [file jci-133-125986-s172.pdf]
